# New Atomistic Insights on the Chemical Mechanical Polishing of Silica Glass with Ceria Nanoparticles

**DOI:** 10.1021/acs.langmuir.3c00304

**Published:** 2023-04-08

**Authors:** Luca Brugnoli, Katsuaki Miyatani, Masatoshi Akaji, Shingo Urata, Alfonso Pedone

**Affiliations:** † Department of Chemical and Geological Sciences, University of Modena and Reggio Emilia, via G. Campi 103, 41125 Modena, Italia; ‡ Innovative Technology Laboratories, AGC Inc., Yokohama, Kanagawa 230-0045, Japan; § Electronics Company, AGC Inc., Yokohama, Kanagawa 230-0045, Japan

## Abstract

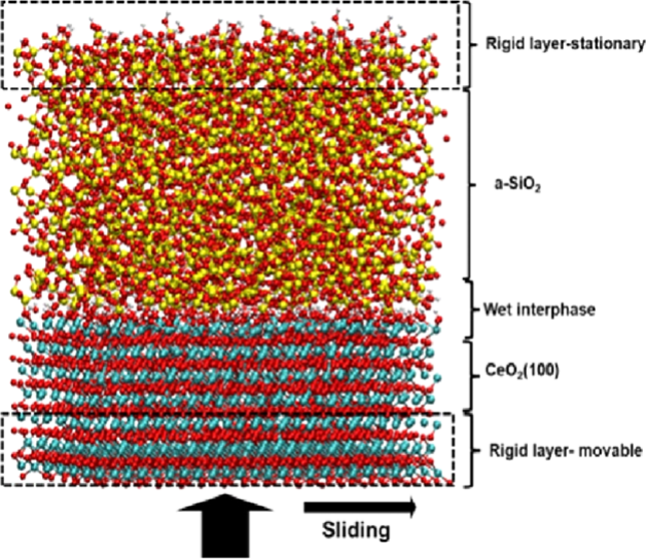

Reactive molecular dynamics simulations have been used to simulate the chemical mechanical polishing (CMP) process of silica glass surfaces with the ceria (111) and (100) surfaces, which are predominantly found in ceria nanoparticles. Since it is known that an alteration layer is formed at the glass surface as a consequence of the chemical interactions with the slurry solutions used for polishing, we have created several glass surface models with different degrees of hydroxylation and porosity for investigating their morphology and chemistry after the interaction with acidic, neutral, and basic water solutions and the ceria surfaces. Both the chemical and mechanical effects under different pressure and temperature conditions have been studied and clarified. According to the simulation results, we have found that the silica slab with a higher degree of hydroxylation (thicker alteration layer) is more reactive, suggesting that proper chemical treatment is fundamental to augment the polishing efficiency. The reactivity between the silica and ceria (111) surfaces is higher at neutral pH since more OH groups present at the two surfaces increased the Si–O–Ce bonds formed at the interface. Usually, an outermost tetrahedral silicate unit connected to the rest of the silicate network through a single bond was removed during the polishing simulations. We observed that higher pressure and temperature accelerated the removal of more SiO_4_ units. However, excessively high pressure was found to be detrimental since the heterogeneous detachment of SiO_4_ units led to rougher surfaces and breakage of the Si–O–Si bond, even in the bulk of the glass. Despite the lower concentration of Ce ions at the surface resulting in the lower amount of Si–O–Ce formed, the (100) ceria surface was intrinsically more reactive than (111). The different atomic-scale mechanisms of silica removal at the two ceria surfaces were described and discussed.

## 1 Introduction

Chemical mechanical polishing (CMP) of silica-based glasses is a key technology for the planarization and the production of defect-free ultrasmooth glass surfaces essential for a wide range of applications, including display panels, flat glass for window panes, optical glasses, precision glass lenses, liquid crystal displays, glass magnetic memory disks, silicon wafers, etc.^1−5^ CMP works with a combination of mechanical abrasion by abrasive particles and simultaneous chemical etching by appropriate chemical additives included in the polishing slurry or the particles themselves.

**Figure 1:**
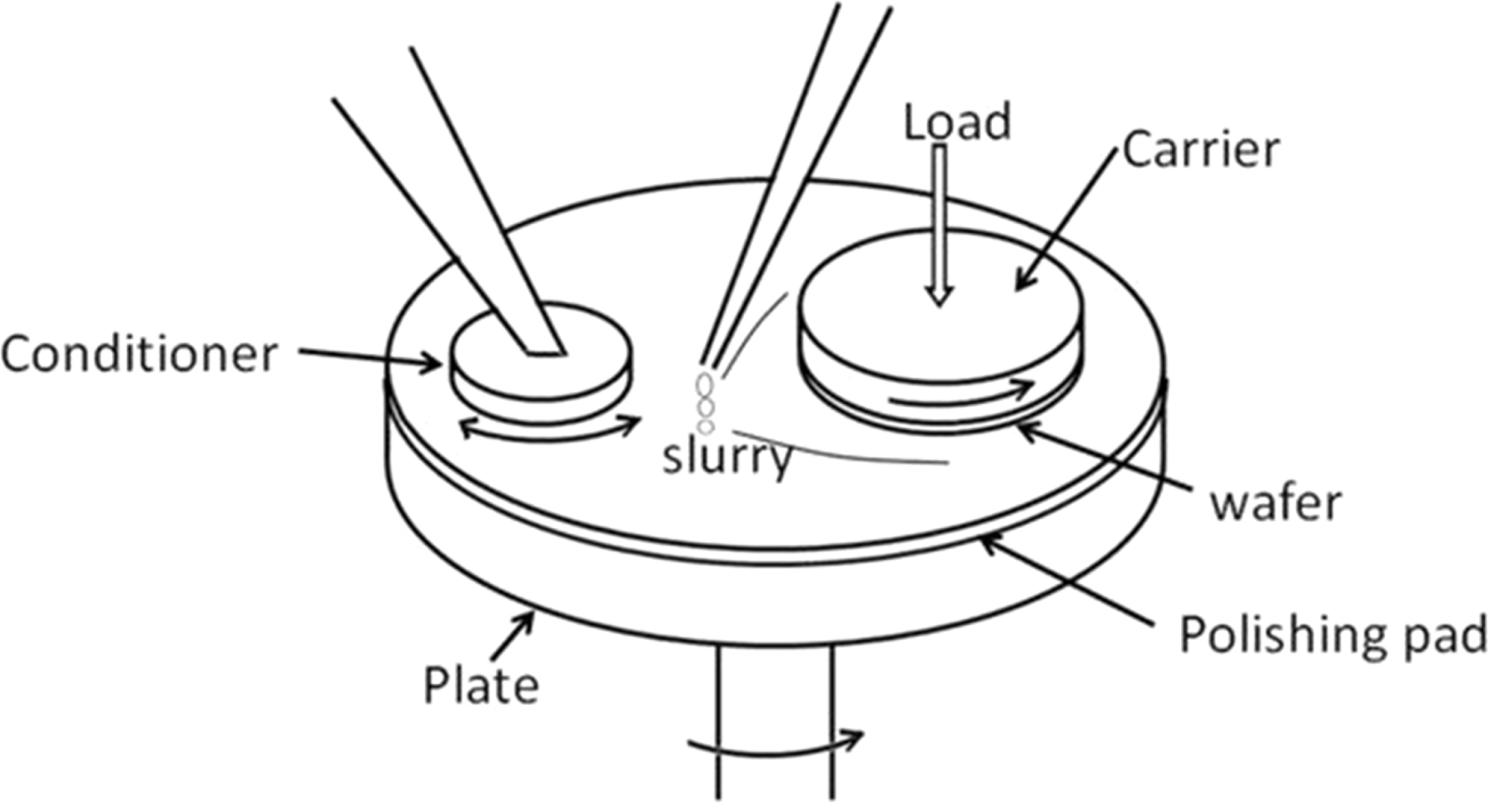
Schematic representation of a typical CMP system. Taken from ref (11), used under the Creative Commons CC-BY license. The abrasive slurry is poured onto the polishing pad and mounted on a rotating plate. The wafer to be polished is held in position by the carrier, which applies a carefully regulated load to control polishing performance. The conditioner cleans the polishing pad, maintaining its effectiveness, using chemical additives or a mechanical tool.

Figure 1 shows a typical CMP system which consists of three main components: a polymeric polishing pad, the slurry with abrasive particles suspended in aqueous solutions, and, of course, the surface to be polished (wafer). During the CMP, the wafer is pressed against the compliant pad, which serves as a conduit for the abrasive particles. The material removal rates (MRR) and surface roughness (SR) can be controlled by judicious selection of particle chemistry, slurry additives, and operation parameters, such as the applied pressure, the slurry flow rate, the speed of rotation, and the time of operation.^4,6−10^

Several studies have been performed to understand the effects of polishing process parameters on final surface quality and a mechanism for how material removal takes place during the polishing process, but still, these aspects need to be understood more in depth.^12−14^ The hardness and size distribution of the abrasive nanoparticles play a very important role in glass polishing, especially for mechanical abrasion.^15^ For instance, the hardness should be similar to that of the glass to avoid deep penetration of the polishing grains and the formation of large grooves at the glass surface. The pressure and rotating velocity of the pad exert the same effect to increase the MRR, while the excess conditions produce rougher surfaces.^10,16^ The chemistry and reactivity of the abrasive nanoparticles, their isoelectric point (the pH at which the surface is neutral), as well as the pH of the slurry and the solubility of the glass in the slurry environment, hugely affect the planarization rate, surface finish, and defectivity of the process. These are all factors that influence the chemical composition of the polishing process through the direct interaction of the particle with the glass substrate.^17^

Independently of the nature of the abrasive nanoparticles, it has been demonstrated that the polishing rate strongly depends on the chemical durability rather than on the hardness and softening point of the glass to be polished,^18^ indicating that chemical reactions influence the glass polishing rate. Moreover, the presence of water is fundamental for glass polishing since the MRR is substantially suppressed when hydrocarbon liquids (kerosene, paraffin, or oil) and nonprotic solvents are used.^17^ In dry conditions or with hydrocarbon liquids, the polishing rates correlate with the hardness of the glass solely since the glass surface does not undergo chemical modifications. Furthermore, mechanical abrasion models often obviously underestimate the MRRs for the polishing conditions with water. Accordingly, Cook^17^ proposed a chemical model for the polishing mechanism dominated by the kinetics of glass dissolution and silica gel formation at the glass surface.

The mass transport during polishing is determined by the relative velocities of the (i) water penetration and diffusion into the surface layer under the load imposed by the abrasive nanoparticles; (ii) hydrolysis of Si–O–Si bonds and dissolution of Si(OH)_4_ molecules in the solution (glass dissolution); (iii) the adsorption of the dissolution products onto the surface of the abrasive nanoparticle, and (iv) the rate of back-deposition on the glass surface forming an alteration gel layer with a range of 1–20 nm thickness.^19^

Among abrasive materials (such as SiO_2_, ZrO_2_, and A1_2_O_3_), ceria (CeO_2_) is the most used due to the highest MRR and the high surface quality.^17^ Abiade et al.^20^ showed that the MRR strongly depends on the pH of the slurry—it increases with an increase of pH in the acidic region and reaches the maximum at neutral pH = 6.8, which corresponds to the isoelectric point (IEP) of ceria, while it decreases when the pH increases in the basic region (pH > 9) due to the agglomeration of ceria nanoparticles. This behavior has been rationalized on the basis of the excess surface charges of silica and ceria at the pH values estimated by zeta potential measurements and adsorption of the orthosilicate ions on the ceria nanoparticles.^21^

This is due to the ability of metal oxides to form the surface functional groups that can act as Brönsted bases and acids when the pH of the solution is below and above the IEP, respectively, as schematized in the following equations:
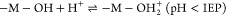
1
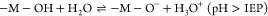
2

During the CMP process at pH = 7, the ceria surface is neutral (IEP = 6.8), whereas the silica glass surface has a net negative charge (IEP = 2). The removal of the materials is thought to occur through the temporary formation of the Ce–O–Si bond when the ceria particle and silica glass come into contact.
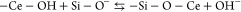
3

It has been estimated that one SiO_4_ unit is extracted every 24 collisions between the ceria abrasive and the silica substrate, whereas 500 million collisions are necessary for the silica abrasives.^17^

Different nanosized CeO_2_ nanoparticles with different morphologies like nanorods, nanocubes, and nanospheres have been synthesized, and their surfaces are tailored to enhance the CMP efficiency. High-resolution transmission electron microscopy (HRTEM) measurements revealed that the (111) and (100) CeO_2_ faces are the more exposed at the surfaces of these nanosystems, whereas X-ray photoelectron spectroscopy (XPS) analysis showed that the CMP efficiency increases with the hydrophilicity of nanoabrasives (and thus with the polar (100) surface) and the concentration of Ce^3+^ on the surface.^4,9^

This is in contrast with the study by Kirk and Wood that observed enhanced polishing efficiency for the ceria particles calcinated at higher temperature (850 °C), which predominantly exposed the facet (111) than those calcinated at a lower temperature (350 °C) to prevail facets (100).^22,23^ Stanek et al. interpreted these findings using hydroxylation energy (*E*_OH_) calculations for the main low-index surfaces of ceria, (111), (110), and (100), at different hydroxylation degrees.^22^ They observed that the hydroxylation energy of the (111) surface was lower than that of the (100) surface at low water coverage, while it became greater at 75% of the coverage ratio, revealing that the equilibrium CeO_2_ crystallite morphology varies depending on the degree of hydroxylation. Based on the calculations, they suggested that if the chemical reaction between CeO_2_ and the glass surfaces involves the transfer of protons or hydroxide species from the CeO_2_ surface to the glass, the (111) surface would be more effective than the other surfaces. This is because the (111) surface does not exhibit a strong affinity for the hydroxide species, and the hydroxyls and protons are thus more easily transferred from the surface (111) to silica, according to the assumed mechanism of the detachment of the monomer Si(OH)_4_.

Another interesting fact is that surface modification with a dispersant such as γ-aminopropyltriethoxysilane (APS) has been found to produce much better surface quality than unmodified ceria particles despite the lower MRR. This was explained by the hardness reduction of ceria particles, the enhancement of lubrication between the particles and substrate surfaces, and the elimination of the agglomerates among the ceria particles occurring at basic pH.^24^

Despite the considerable amounts of investigations carried out and the plausible mechanisms proposed above, an atomistic-level understanding of the CMP process of the silicate glass surfaces with the ceria nanoparticles is still lacking. Since CMP is a dynamic process between the two materials, the inaccessible buried surface is difficult to be studied by *in situ* experiments. Although atomic force microscopy (AFM) and TEM measurements have been employed to find the possible chemical reactions involved in the CMP processes by observing the chemical species on the substrate surface before and after the CMP processes, they cannot clarify the effects of mechanical force on the chemical reactions as well as the dynamic mechanisms.

Conventional classical molecular dynamics (MD) simulations have been successfully applied in simulating the mechanical process in CMP,^25^ while they failed in describing the chemical reactions involved in the CMP processes. On the other hand, *ab initio* MD simulations provided useful insights into the tribological processes at the solid–solid and solid–liquid interfaces, but the application is limited only to investigate small systems in a very short time.^26^ Indeed, a few *ab initio MD* simulations studied the ceria silica interactions using small ceria clusters (H_4_Ce_6_O_12_) interacting with model α-quartz surfaces for a few picoseconds.^27−29^ These theoretical studies demonstrated that the direct interaction of the ceria cluster with the dry silica surface first promoted the dissociation of the Si–O_Si_ and Ce–O_Ce_ bonds, and subsequently, a Si–O–Ce bond was formed. Additionally, because the oxygen defects and the associated Ce^3+^ sites promoted the reaction with hydroxylated surfaces and elongated the Si–O bonds at the surface, the siloxane bond was dissociated due to the subsequent attack by a water molecule. These reactions were suggested to soften the silica glass surface and then to promote mechanical polishing.

In recent years, the development of reactive force-fields^30^ (e.g., ReaxFF), which are able to describe bond-breaking and bond-forming processes, has made possible the investigations of several important chemical phenomena comprising the CMP processes of semiconductors (like silicon) used in the integrated circuits and the silica substrates with an abrasive in water solutions. These studies demonstrated the applicability of the reactive force-fields in elucidating the interaction between chemical and mechanical effects.^31,32^ In the early studies, the polishing efficiency was measured by counting the number of siloxane bridges formed, and the bridges were found to increase with the pressure applied at the interface and with the addition of H_2_O_2_, which increases the oxidation state of the surfaces (higher degree of hydroxylation).^33,34^

Although the ReaxFF MD simulations have been conducted to study the reactivity of silica and sodium silicate glasses and the formation of the alteration silica gel on the glass surfaces when interacting with water solutions,^35−38^ to the best of our knowledge, a study investigating ReaxFF MD simulations on silica removal by ceria abrasives has never been reported. This is probably due to the lack of appropriate Si/O/H/Ce parameters in ReaxFF. To get new atomistic insights into this important process, we have developed ReaxFF parameters for simulating silica/ceria/water interactions by maintaining compatibility with the previous parameter set for silica and water developed by Fogarty et al.,^39^ and later reparametrized by Yeon and van Duin^40^ to reproduce the silicon hydroxylation barrier and then further extended by Hahn et al.^41^ to describe sodium silicate systems.

The aims of this work are to investigate (1) the effects of the glass surface morphology and the gel layer formed by hydroxylation; (2) the process of silica extraction as isolated SiO_4_ units dimers or chains and relating phenomena, such as Ce–O–Si bond-forming and anchoring sites required to extract them; (3) difference of the ceria surface morphologies, (111) *vs* (100); (4) effects of the hydroxylation and hydration degrees in relation to the pH of the slurry on the CMP efficacy; and (5) how the atomic level mechanism changes with loading pressure and temperature.

## 2 Computational Methods

### 2.1 ReaxFF Parameterization

All MD simulations were performed using LAMMPS software^42^ with ReaxFF.^30^ ReaxFF has been developed to simulate reactions by fitting the parameters to reproduce the results of the *ab initio* calculations. The substantially lower computational cost compared with *ab initio* calculations enables to model of larger systems with up to millions of atoms.^43^ ReaxFF adopts a bond-order dependent description of the two-, three-, and four-body interactions, which mimic the quantum mechanical nature of the chemical bond, allowing it to simulate reactive events such as the formation and dissociation of chemical bonds. The bond order of all atomic species in a molecular system is updated at each iteration of the simulation to evaluate energy and forces, which should continuously change with time. In addition, the nonbonding van der Waals and Coulombic interactions are considered. The van der Waals interactions are computed according to a screened Morse potential. The partial charges of atomic species to evaluate the electrostatic interaction are computed using the electronegativity equalization method (EEM).^44^

ReaxFF was originally developed for simulating hydrocarbon systems and was then extended to study proteins, semiconductors, metals, oxides, silicates, and so on.^45^ In this study, the interactions among the species Si/O/H/Ce/Na/Cl were considered. Most of the parameters were available from previous works. For instance, the parameters for Ce/O/H were taken from our previous study on the ceria/water interactions,^46^ while those for Si/O/H/Na were from Hahn et al.,^41^ which studied the interaction between sodium silicate glass and water. The Si/O/H/Na/Cl parameter set was composed of the Na/Cl/O/H parameters developed for studying alkali halides in water by Fedkin et al.^47^ and the parameters for O/H in second-generation ReaxFF for water, which better describes the liquid phase.^48^ Contrary to the available parameter sets, ReaxFF parameters for the two-body interactions between Ce and Si and the three-body interactions for Ce–O–Si were newly developed in this work. In this study, we also modeled NaOH and HCl solutions, but the bonding interactions of Na–Ce and Cl–Ce were not considered. This is because the two cationic species, Na^+^ and Ce^4+^, would not form bonds, while for Cl^–^ and Ce^4+^, we assumed only electrostatic and van der Waals interactions. The details of the parameterization procedure, the quantum mechanical datasets, and the Ce/O/H/Si/Na/Cl ReaxFF library (in LAMMPS format) are reported in the Supporting Information (SI).

### 2.2 Amorphous Silica Models

To investigate the CMP process, we first adopted slab-on-slab structures to model the CeO_2_/SiO_2_ glass interface by contacting a flat (111) or (100) CeO_2_ surface with three types of amorphous silica glass models. Two sets of silica glass models with a size of 4.5914 nm × 3.9763 nm × 4.3000 nm and 4.3280 nm × 4.3280 nm × 4.1910 nm were constructed for simulating the interaction with the flat ceria (111) and ceria (100) models, respectively. Both the silica glass models were composed of 1728 silicon atoms and 3456 oxygen atoms with a density of 2.20 g/cm^3^. Initially, these atoms were randomly placed in the box by avoiding interatomic distances shorter than 2.2 Å. The glass structures were then generated using a melt-and-quench approach.^49^ The MD simulations were first performed at 4000 K for 50 ps; then, the system was cooled down to 300 K with a cooling rate of 2 K/ps. The system was then further equilibrated at 300 K for 50 ps. A timestep of 0.5 fs was used for the integration of atom motions. During the melt–quench simulations, we disabled the bonding interactions between O atoms to avoid the formation of gaseous O_2_ at temperatures higher than 2000 K, as adopted in previous studies.^50^

From the bulk silica glass models, the surface models were generated by truncating the periodic boundary condition along the *z*-axis, resulting in two slabs of thickness 43 and 41.91 Å, suitable to match with the 111 and 100 ceria models, respectively. A vacuum region with a 3 nm thickness was added between the terminations of the silica glass slab models, and the space was filled with 1880 water molecules.

Since a silica gel forms at the glass surface during the CMP process,^51^ we created other two models of silica surfaces possessing a thin silica gel layer. The silica gel is supposed to be formed by the hydration of the silica surface, hydrolysis of siloxane bonds, dissolution of orthosilicic units, and subsequent condensation reactions at the glass surface. However, the timescale of the gel formation is too long for nanoscale classical MD simulations; therefore, we artificially generated a 5 Å thick gel layer by adopting a method proposed by Du and Rimsza.^38,52^ In this method, a specific fraction of silicon atoms was randomly removed from the outer layer of the silica slab models, and the resultant dangling bonds on the oxygen atoms were saturated with hydrogen atoms. To understand the effect of the degree of silica gel polymerization, we built two gel models by removing 20 and 40% of Si atoms. These gel models are abbreviated as Gel20% and Gel40%, respectively, while the original silica glass model is noted as Gel0%, hereafter. After the formation of the initial silica gel models, we performed equilibration simulations for 500 ps in water at 300 K.

### 2.3 Ceria Models

The ceria slab models with the (100) and (111) surfaces were generated by cleaving a crystalline bulk model of ceria (*a* = 5.401 Å). The (100) surface model was composed of 12 atomic layers with 8 × 8 unit cells, which corresponds to a surface lattice of 4.32 nm × 4.32 nm size. Since this surface is polar, half of the O atoms were moved to the other side, as in the case of a previous work.^46^ In contrast, the (111) surface possessed 18 atomic layers with an unconventional rectangular lattice of 4.5914 nm × 3.9763 nm instead of the conventional hexagonal structure. The ceria surfaces of the slab models were equilibrated in contact with different pH solutions. To make the acidic and basic solutions, 10 HCl and 10 NaOH molecules were added to water, respectively. The nominal pH values of the acid and basic solutions are 0.5 and 13.5, respectively.

### 2.4 Setting the Slab-On-Slab Model and the Polishing Procedure

**Figure 2:**
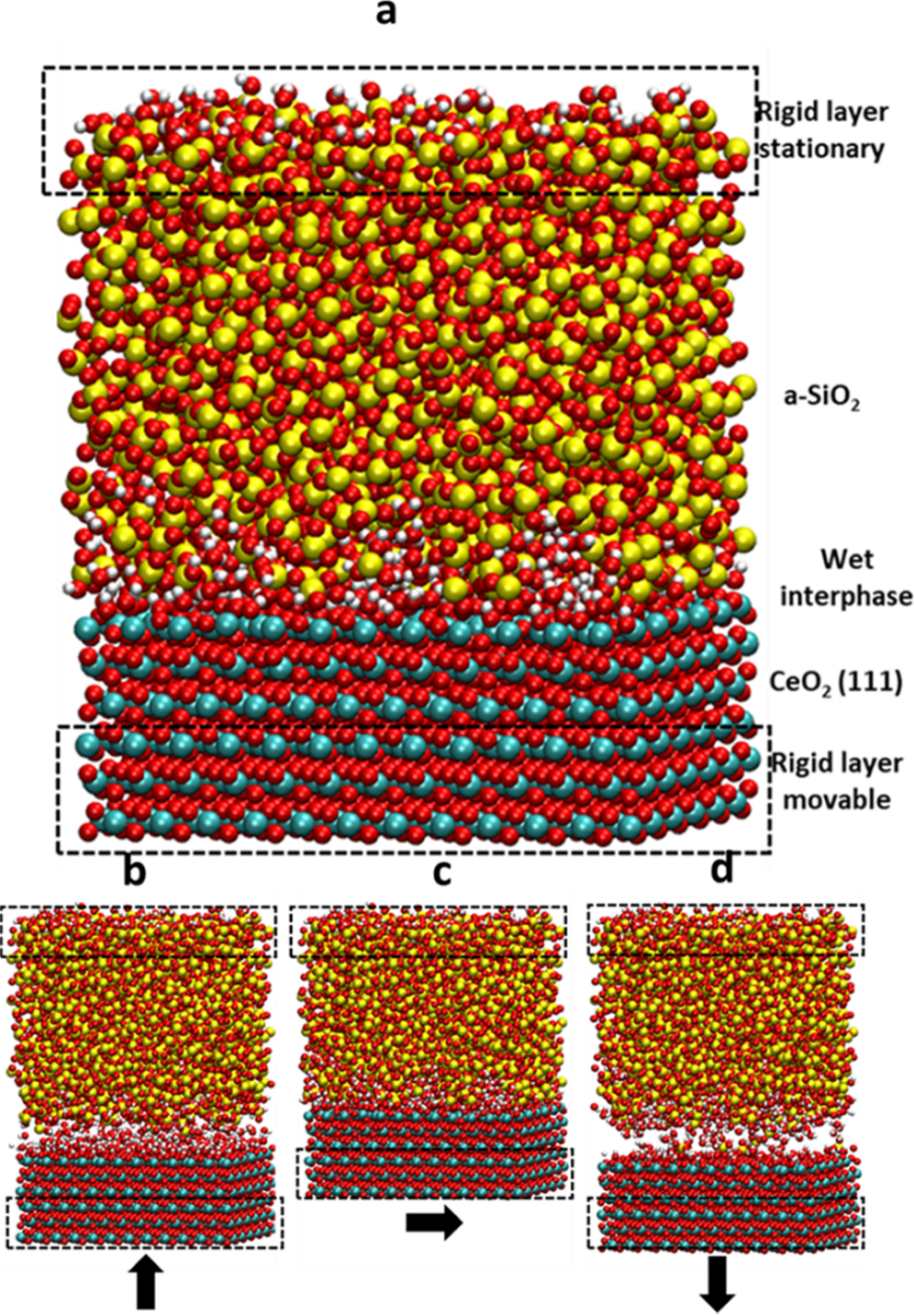
Schematics of the chemical mechanical polishing model composed of amorphous silica and cerium oxide in an aqueous environment (a). The polishing simulation is performed in 3 phases, where the rigid bottom layer of ceria is moved toward the silica surface during the compression (b), then it is moved parallel to the interphase during the sliding (c), and finally, it is moved away from the silica during the detachment phase (d).

To investigate the polishing process, the ceria slab models were placed under the silica glass models to form a multilayer slab model. A limited number of water molecules were put between ceria and silica glass models. Figure 2a shows the model adopted for simulating the chemical mechanical polishing of the silica surface (Gel0%) with the ceria surface (100) model as an example.

The polishing simulation was composed of three steps. First, the system was compressed by pushing the ceria model toward the silica glass model with a constant speed *v_z_* of 1 m/s until the pressure normal to the interface (*P_zz_*) reached the target one (Figure 2b). Second, the ceria slab model was moved along the *x* direction with a constant speed *v_x_* of 10 m/s for 450 ps (Figure 2c). After the sliding simulation, the two surfaces were separated by moving the ceria slab away from the silica glass slab with a speed *v_z_* of −1 m/s (Figure 2d). In the models adopted for polishing, the silica atoms at the top (7 Å) of the slab model reported in Figure 2a are treated as a rigid body and kept frozen for all of the simulations. The same has been done for the atoms at the bottom of the ceria slab (8 Å). This implies that the relative positions of the atoms in each group remain constant throughout the simulations and that the forces acting on them are null. The silica termination was kept frozen, while a constant translational speed was assigned to the ceria rigid termination. This was needed to exert a mechanical action on the rest of the system.

It is essential to emphasize that the moving rigid slab exerts a mechanical action on the rest of the system, which responds by being compressed, dragged, and pulled according to the different polishing steps. In this work, the speed is assigned to the rigid block of atoms by using the function “move” and the option “linear” of LAMMPS, which overrides the speeds derived from the integration of the motion equation. To avoid issues, all of the frozen atoms and those with an assigned speed are excluded from the equation of motion. Nevertheless, these atoms kept exerting forces on all of the other atoms through the force field short- and long-range interactions. All of these polishing simulations were conducted with a shorter timestep of 0.25 fs to integrate the equations of motion using the Verlet algorithm.

## 3 Results and Discussions

### 3.1 Chemistry and Morphology of the Surface Models for Silica and Ceria before CMP

**Figure 3:**
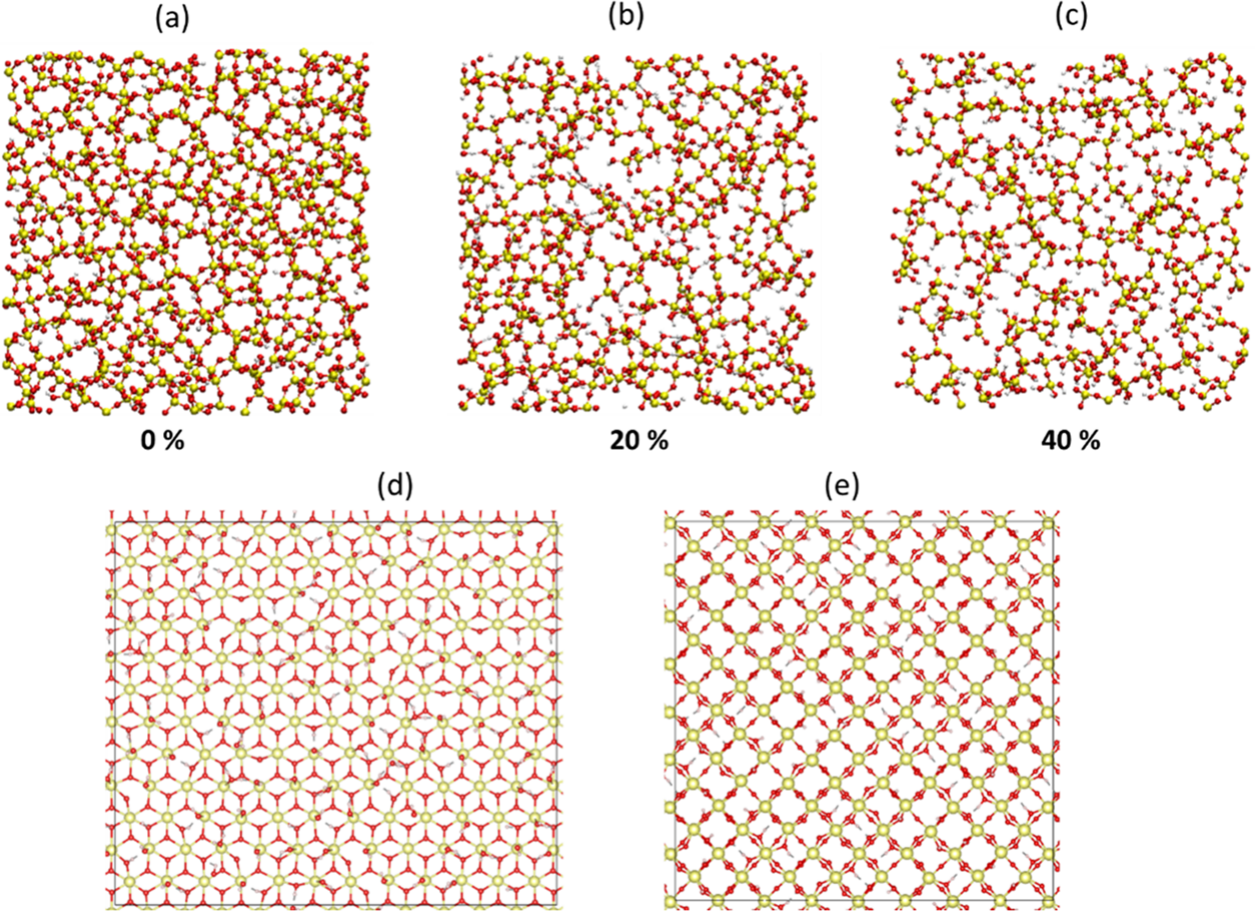
Top views of the outermost layers for the silica surface models of (a) Gel0%, (b) Gel20%, and (c) Gel40% and (d) ceria (111) and (e) (100) surfaces after equilibration in neutral water.

**Figure 4:**
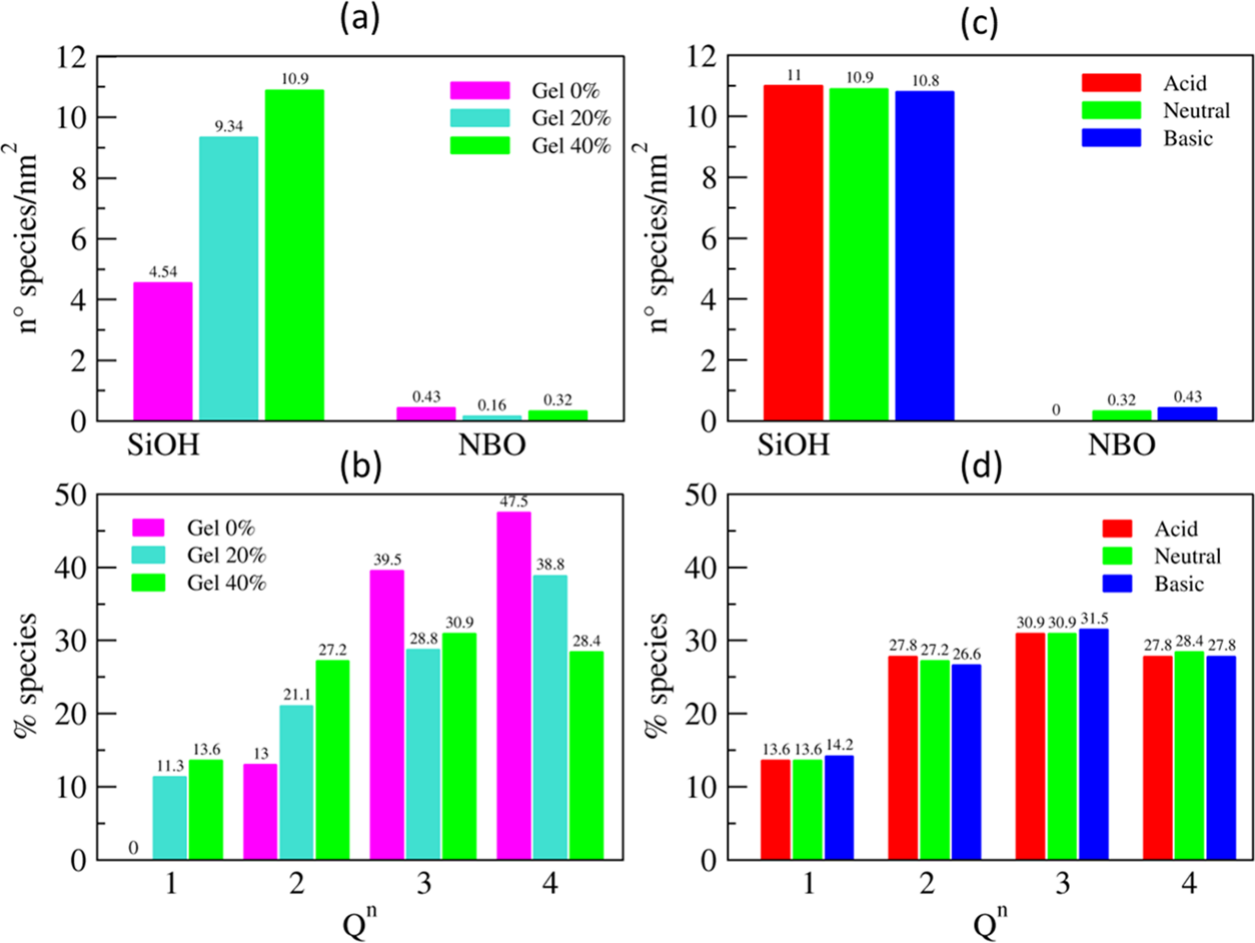
(a) Concentrations of the silanols and NBO species and (b) *Q^n^* distributions of silicon in the outermost surface layer of a 5 Å thickness of silica glass. (c) Silanols and NBO concentrations and (d) *Q^n^* distributions for the Gel40% models in the acidic, neutral, and basic solutions.

The slab models composed of silica glass and ceria were first analyzed to understand the structural characteristics before polishing simulations. Figure 3 shows the surfaces of the three silica glass models (Gel0%, Gel20%, and Gel40%) and the hydroxylated (100) and (111) surfaces of ceria. Figure 4 reports (a) the concentration of silanols and (b) the distribution of *Q^n^* species (*Q* stands for quaternary Si species and *n* is the number of bridging oxygens (BO) on them) at the outermost surface with a 5 Å thickness after equilibration in neutral water.

The concentration of surface silanols for the Gel0% model was 4.54 ± 0.03 Si–OH nm^–2^, which is in good agreement with the experimental value of 4.6 Si–OH nm^–2^ for the fully hydroxylated silica glass and those estimated by previous ReaxFF simulations.^53,54^ The silanol concentrations at the Gel20% and Gel40% surfaces were 9.34 and 11.05 Si–OH nm^–2^, respectively. Figure 4b shows that the polymerization of the silica network decreased with increasing the degree of jellification. Indeed, the Gel0% model exhibited more *Q*^3^ and *Q*^4^ species and more porous structures mainly composed of *Q*^1^ and *Q*^2^ species were formed for the Gel20% and Gel40% models. Consequently, the degrees of the network connectivity within the outermost surface of 5 Å thickness were 3.34, 2.95, and 2.74 for the Gel0%, Gel20%, and Gel40% models, respectively.

The effect of pH on the surface morphology was examined using the Gel40% model, as shown in Figure 4c,d. It was found that more silanols were formed in the lower pH solutions; it increased from 10.9 nm^2^ in the neutral environment to 11.0 nm^2^ in the acid solution, while it decreased to 10.8 nm^–2^ in the alkaline solution. The variability in these data determined by producing three silica models is 0.1 nm^–2^. Consistently, the surface nonbridging oxygens (NBO) increased with the increase of the pH of the solutions due to the deprotonation of the oxygen atoms at the glass surface.

Additionally, the variation of the solution pH was found to affect the network connectivity of silica, albeit to a limited extent: with respect to the neutral case, the network connectivity decreased from 2.74 to 2.72 in both the acid and basic environments. In both cases, this was ascribed to the hydrolysis of a siloxane bond between *Q*^4^ and *Q*^3^ species in the acidic condition to form *Q*^3^ and *Q*^2^ species and between *Q*^4^ and *Q*^2^ species in the basic condition to form *Q*^3^ and *Q*^1^ species. In fact, as can be observed in Figure 4d, in an acidic environment, the % of *Q*^4^ decreases from 28.4 to 27.8, while *Q*^2^ increases from 27.2 to 27.8, while there is no change in the % of *Q*^3^. In the basic environment, the % of *Q*^4^ and *Q*^2^ decreased, respectively, from 28.4 to 27.8 and from 30.9 to 31.5, while the % of *Q*^3^ and *Q*^1^ increased, respectively, from 28.4 to 27.8 and from 13.6 to 14.2. This is naturally expected by the depolymerization of the silica network, which is catalyzed in both acid and alkaline conditions. As stated by Rimsza et al.,^52^ the time frame of this process is too long to be fully simulated by adopting a conventional molecular dynamics protocol; hence, the artificially depolymerized models were employed.

**Figure 5:**
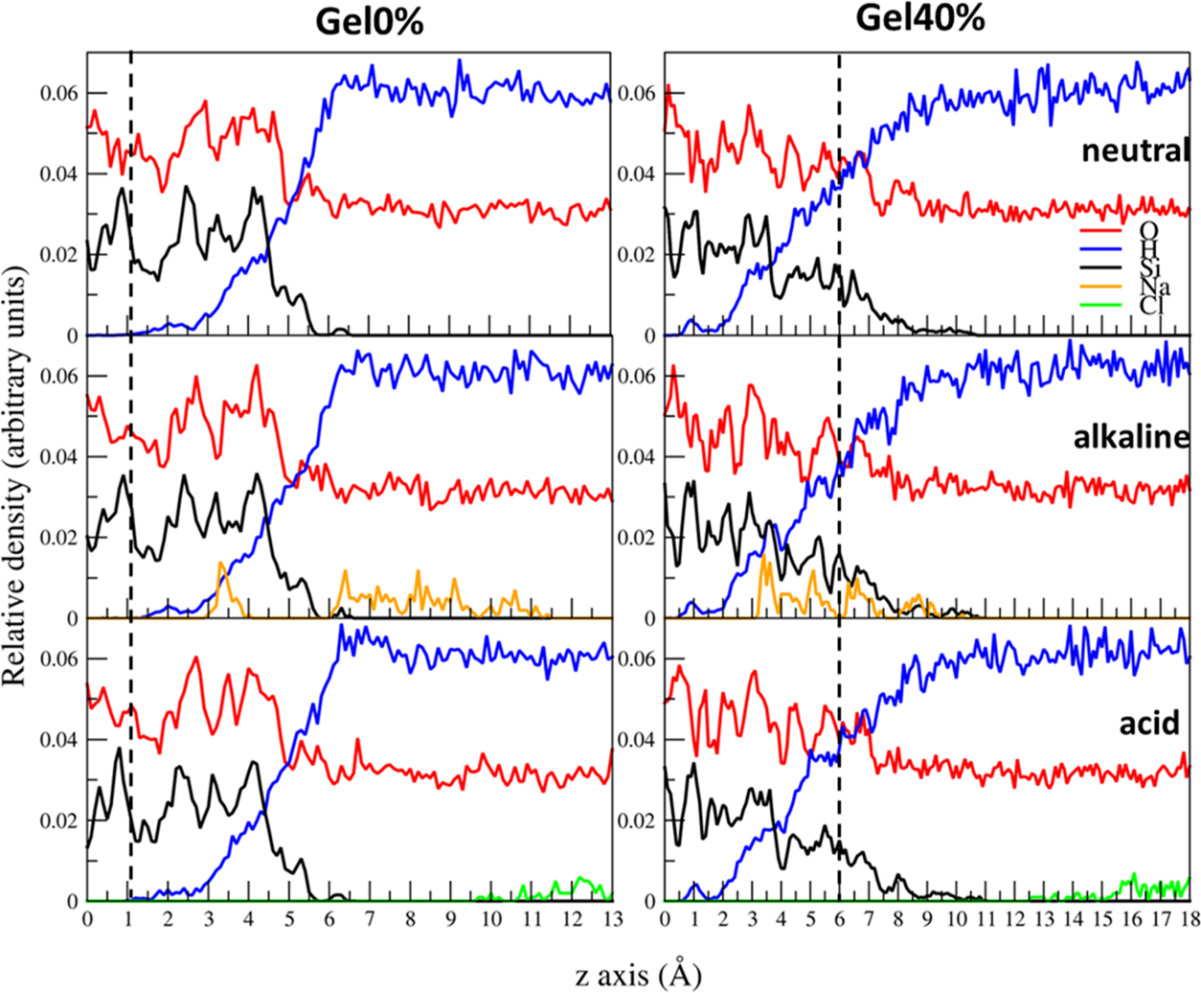
Atomic density profiles along the z-axis of the Gel0% and Gel40% models equilibrated at different pH. The vertical dashed lines represent the boundary between the gel layer and bulk silica which has a thickness of 5 Å.

**Figure 6:**
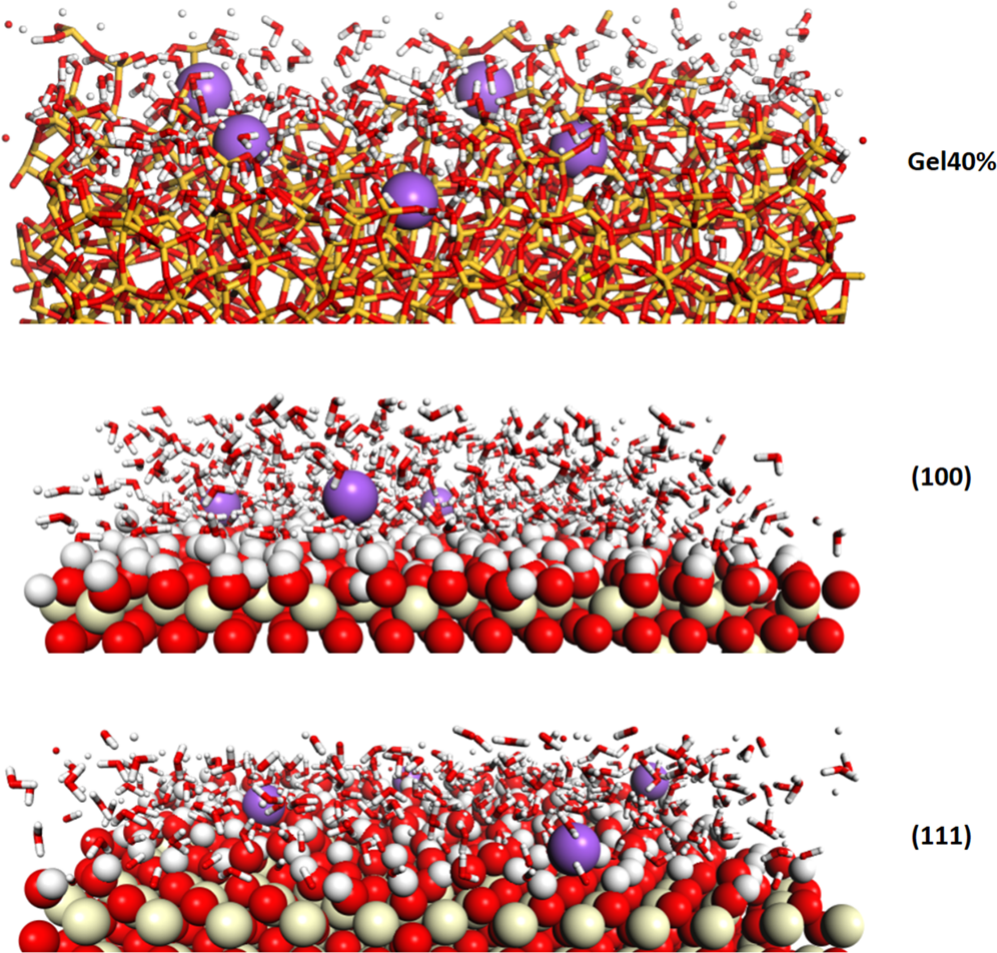
Views of the Gel40%, CeO_2_ (100), and (111) surfaces equilibrated in an alkaline environment showing adsorption and penetration (in the case of Gel40%) of Na^+^ ions (violet spheres).

The z-profile concentrations of the atomic species for the Gel0% and Gel40% models at different pH are shown in Figure 5. It is interesting to note that in the alkaline environment, the majority of the Na^+^ ions adsorbed at the glass/water interface, indicating that the silica surface was negatively charged in the basic solution. This phenomenon has also been observed at an interface between the NaOH solution and silica surface in the previous ReaxFF MD study.^55^ Our simulations revealed that Na^+^ ions penetrate into the porous alteration layer of the Gel40% model, while the penetration of Na^+^ ions was inhibited for the Gel0% model, as shown in Figure 6. In the alteration gel layer, Na^+^ ions were often sixfold-coordinated by three silanols, a BO, and two water molecules, whereas those adsorbed on the Gel0% surface were usually coordinated by three silanols and three water molecules. In contrast, in an acidic environment, the Cl^-^ ions remained in the solution far from the glass surface.

**Figure 7:**
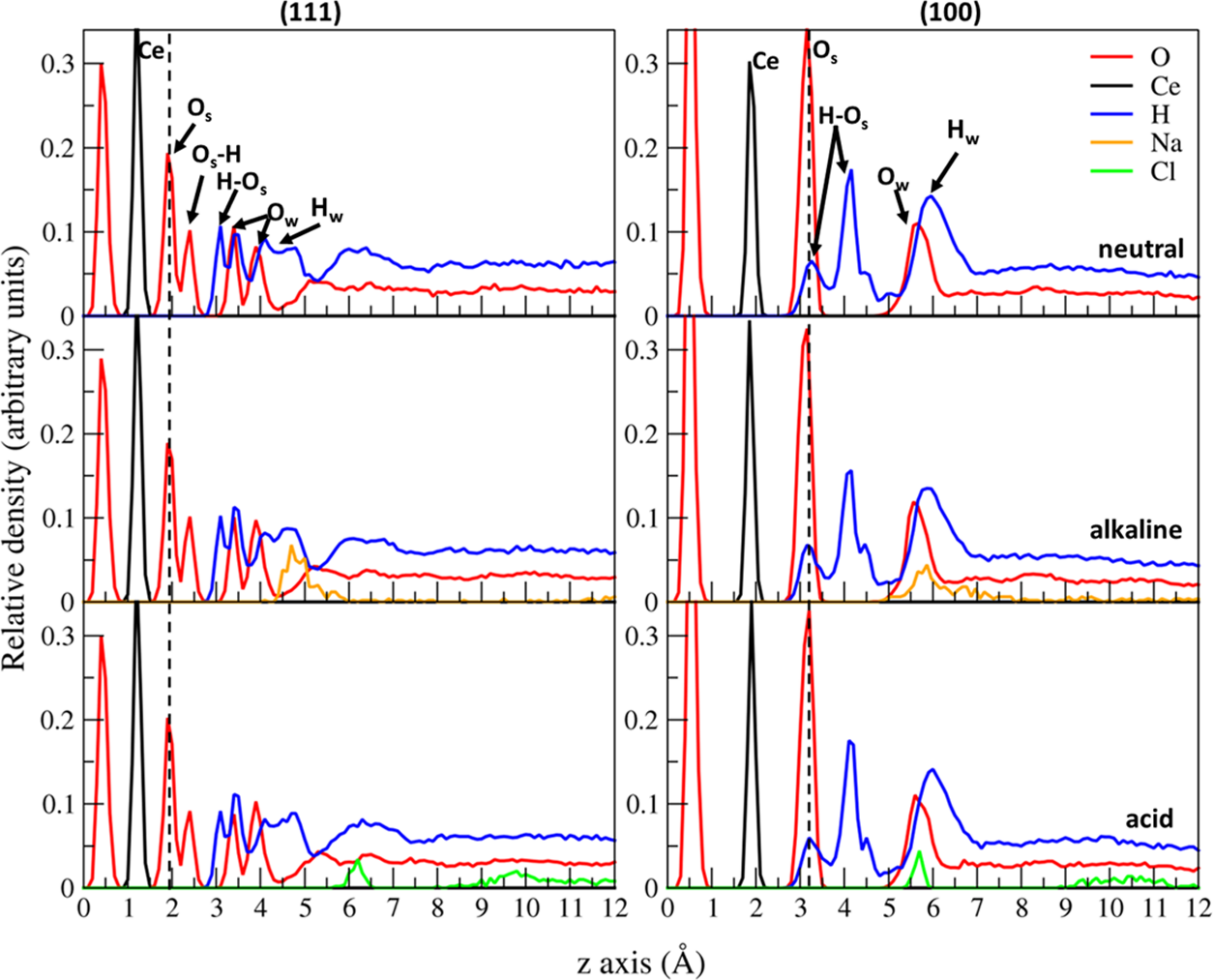
Density profiles of the atomic species (Ce, O, H, Na, and Cl) along the z-axis for the two ceria surfaces equilibrated at different pH. The vertical dashed line represents the ceria outermost layer of oxide oxygens (O_S_). O_S_–H is hydroxylated oxide oxygen, O_W_ = water oxygen, H–O_S_ = hydrogen of hydroxylated O_S_, and H_W_ = water hydrogen.

Figure 3d,e shows the hydroxylated models for the ceria (100) and (111) surfaces equilibrated in neutral water, and the density profiles of the atomic species along the z-axis are shown in Figure 7. The dashed lines in the figures represent the positions of the outermost oxygen atoms (O_S_) of the ceria models.

On the ceria (111) surface, the dissociation of water leads to the protonation of superficial oxygen atoms (Os) connected to three Ce ions and the hydroxylation of Ce in the subsurface layer forming Ce–OH species, as in the case of our previous work, which investigated the structure and dynamics of the ceria/water interface.^46^ In the neutral solution, the populations of Os–H and Ce–OH were almost equal and approximately 2.62 nm^–2^ for each (5.24 nm^–2^ in total), which corresponds to 33% of the available oxygen sites at the surface.

On the one hand, the concentration of molecularly adsorbed water molecules (Ce–OH_2_^+^) was 1.11 nm^–2^ within the first coordination layer. In the acidic solution, the concentrations of O_S_–H and Ce–OH_2_^+^ increased to 2.68 nm^–2^ and 1.32 nm^–2^, respectively, while the concentration of the Ce–OH sites decreased to 2.43 nm^–2^.

The concentrations of Os–H, Ce–OH, and Ce–OH_2_^+^ in the alkaline solution were 2.42 nm^–2^, 2.70 nm^–2^, and 1.16 nm^–2^, respectively. According to these analyses, the highest surface concentration in the sum of O_S_–H and Ce–OH was found at neutral pH, which is close to the isoelectric point of ceria (pH 6.8), and the total concentration decreased at higher and lower pH. This observation agrees with the experimental fact that the MRR is largest at pH 6.8 because the more OH groups at the surfaces promote more Si–O–Ce bonds at the ceria/silica interface during polishing.

The dissociation of water facilitates the protonation and hydroxylation of the O_S_ and Ce sites, as described in our previous work.^46^ Consequently, the surface concentration of hydroxyls was 10.1 nm^–2^ for the CeO_2_ (100) surface in the neutral solution. The density profiles of the oxygen and hydrogen atoms reported in Figure 7 exhibit more intense and structured peaks at the (100) surface in comparison with the (111) surface, implying that the higher hydroxylation (90% of the available sites are occupied) caused more ordered interface structures with the denser hydrogen-bond network, as described in ref (46) Similar density profiles of the atomic species along the z-axis were observed in the acid and basic solutions.

In the alkaline solution, the surface hydroxyl concentration on the ceria decreased to 9.7 nm^–2^ because several Ce–OH groups were deprotonated by the reaction with the excess hydroxide ions in the solution. The surface hydroxyl concentration in the acidic solution was approximately 9.8/nm^2^, which is also lower than that in the neutral solution. A possible reason is that the excess hydronium ions proceeded with protonation of the Ce–OH groups to form Ce–OH_2_^+^, which released water molecules in the solution. Both the ceria surfaces are amphoteric by reacting with either the excess hydroxide or hydronium ions in the solutions.

By assuming formal charges for the elements, we approximately estimated the total charges of the ceria surfaces at the three pH conditions. The overall charge of the ceria (111) surface was zero in the neutral solution, which is in accordance with the fact that the experimental isoelectric point of CeO_2_ is pH = 6.8. In the acidic solution, excess H_3_O^+^ in the solution leads to a positive charge of +5 |*e*^–^| (0.270 |*e*^–^|/nm^2^). In the alkaline solution, excess hydroxide reacts with surface Os–H, leading to a negative charge of −5 |*e*^–^| (−0.270 |*e*^–^|/nm^2^). The negatively charged surface attracted Na^+^ ions, as shown in Figure 6.

On the more reactive ceria (100) surface, the charge density was 0.59–0.64 |*e*^–^|/nm^2^ at neutral pH, whereas it increased to 0.75 |*e*^–^|/nm^2^ in the acidic solution and decreased to 0.43 |*e*^–^|/nm^2^ in the basic solution. The charge on ceria (100) is less than that on the (111) surface at basic pH, but it is still positive. Indeed, the density profiles of Na^+^ ions demonstrated that these ions were farther away from the ceria surfaces and above the first monoatomic layer of the hydroxyls (Ce–OH) due to the repulsive interaction with the positively charged surfaces (see Figure 6). Figure 6 also shows that on the (111) surface, the Na^+^ ions directly interact with the free O_S_ sites (not hydroxylated), in contrast. Accordingly, it was demonstrated that the less reactive (111) surface underwent a more drastic variation in the surface charge from positive to negative by changing the pH, which is in good agreement with zeta potential measurements.^9^ Similarly, the negative Cl^-^ ions were attracted to the positively charged surface, but they did not directly interact with the surfaces and remained around the second hydration layer (see Figure 7). On the ceria (111) surface, the Cl^-^ ions were farther from the surface in comparison with the Na^+^ ions, which penetrated into the first hydration layer and directly interacted with the surface, contrary to ceria (100).

### 3.2 Chemical Mechanical Polishing

#### 3.2.1 Effect of the Silica Model

In this section, we investigate the effect of the silica gel layers formed at the glass surface on the morphology and polymerization when they are in contact with water. Furthermore, the influence of the alteration layers on the material removal efficiency of a ceria (111) surface was examined in the neutral pH condition at 300 K and 5 GPa. To perform the simulations, after pushing the three silica models (Gel0%, Gel20%, and Gel40%) on the ceria (111) surface, the ceria model was slid along the *x* direction for 450 ps.

**Figure 8:**
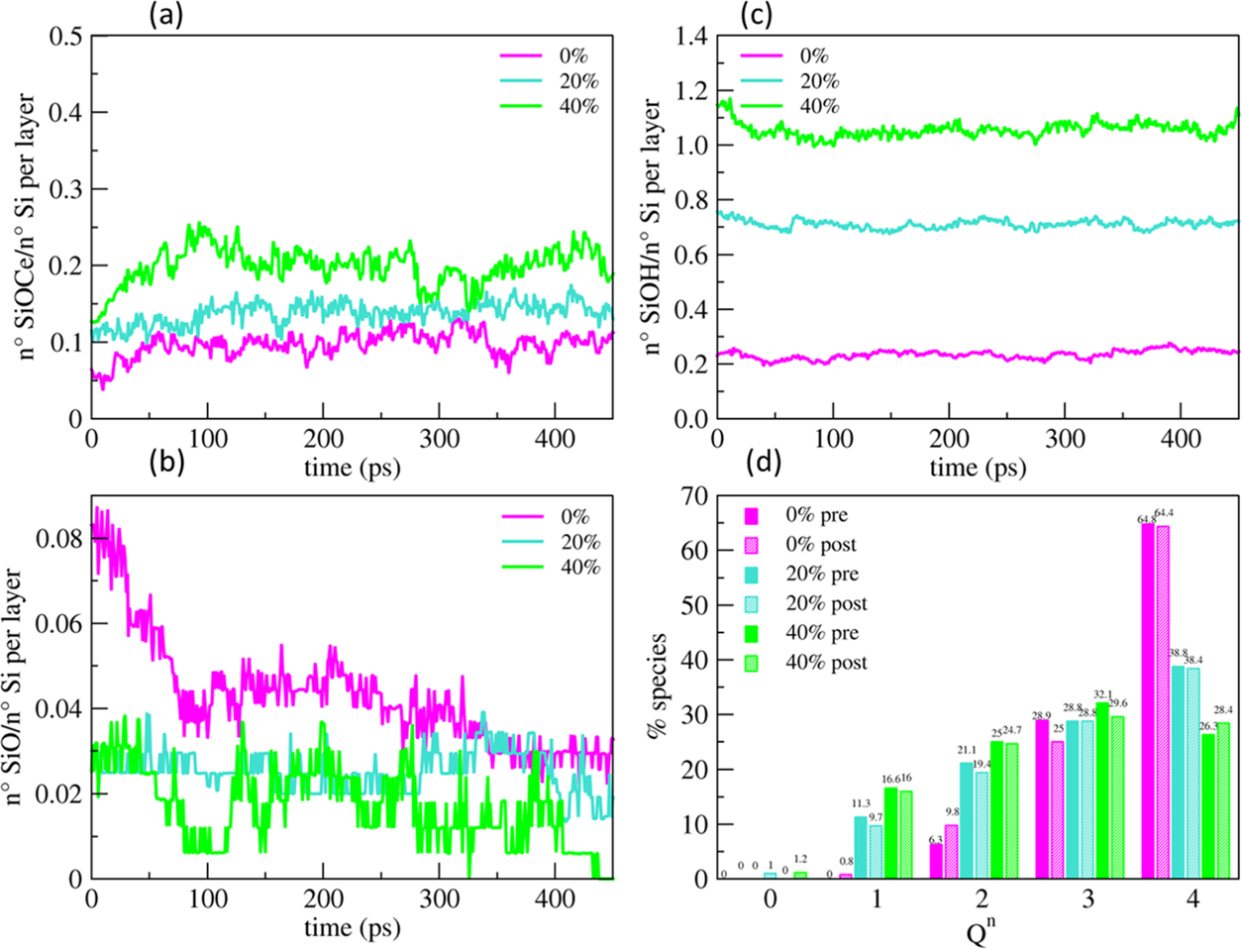
Evolution of the Si–O–Ce bonds (a), silanols (b), and NBOs (c) per silicon atoms in the gel layer (outermost 5 Å) of the three models (Gel0%, Gel20%, and Gel40%) during 450 ps of polishing at 300 K and 5 GPa. (d) Comparison between the *Q^n^* distribution of silicon atoms in the outermost gel layers in the three models before and after polishing.

Figure 8 reports the temporal evolutions of the number of the Si–O–Ce bonds (panel a), silanols (b), and NBOs (c) normalized with respect to the number of silicon atoms at the outermost surface of the 5 Å thick silica layer during polishing. We highlight that the number of the Si–O–Ce bonds in the plot does not start from zero since most of them were formed during the compression step performed before the sliding simulations. More Si–O–Ce bonds per silicon atoms were formed in the Gel40% model compared with Gel0%, which is associated with the number of silanols per silicon atom formed in these models, as reported in Figure 8b. The Gel40% model possessed the largest amount (> 1) of OH groups per silicon atom; therefore, the model was connected with the ceria surface by forming more Si–O–Ce bonds. Figure 8a reveals that the number of Si–O–Ce bonds per silicon increased in the first stage (t <100ps) of the polishing and then fluctuated and finally converged to approximately 0.1, 0.15, and 0.2 Si–O–Ce bonds per silicon for the Gel0%, Gel20%, and Gel40% models, respectively. Simultaneously, both the silanols and NBO species per silicon atoms decreased, suggesting that the Si–O–Ce bonds were produced by both the following reactions
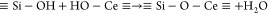
4
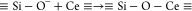
5where Ce≡ represents an undercoordinated cerium atom at the ceria (111) surface. We remind that at this surface, 33% of the Ce sites were hydroxylated and the rest of them were free or solvated by molecular water, which might be pushed away by the Si-NBO or Si–OH groups when silica was compressed and slid on the ceria surface.

Figure 8d reports the ratios of silicon *Q^n^* species in the outermost 5 Å thick layers of the bulk silica and the gel models after polishing. This analysis reveals that after 450 ps sliding of the ceria/silica interfaces, the network connectivity decreased from 3.58 to 3.53 for the Gel0% model, from 3.14 to 2.97 for Gel20%, and from 2.74 to 2.68 for Gel40%. As expected, the Gel0% model is the least reactive due to the densest structure with the highest degree of reticulation and the lowest concentration of silanols. Indeed, any removal of the silica material was not observed in the timescale of the simulations. Contrarily, for the other two models, we observed the removal of 2 isolated orthosilicic units (*Q*^0^ species), which corresponds to 1% of silicon atoms present in the outermost silica layers. In these conditions, namely, neutral pH, 300 K, and 5 GPa, it seems that only one SiO_4_ unit can be removed at a time.

#### 3.2.2 Effect of the pH, Temperature, and Pressure

##### 3.2.2.1 Effects of the pH

The effect of pH on the chemical mechanical polishing of the silica glass with the (111) ceria surface was investigated using the Gel40% model. Employing the gel model is a bold approximation when studying the pH effect since the pH condition is known to affect the kinetics of dissolution and thus the formation and morphology of the alteration layer. However, as stated above, the gel layer formation is outside the capabilities of conventional MD simulations. Therefore, in this section, we study the effect of pH on polishing by employing the layers with the same degree of alteration but differing in the amounts of SiOH, CeOH, and deprotonated groups.

**Figure 9:**
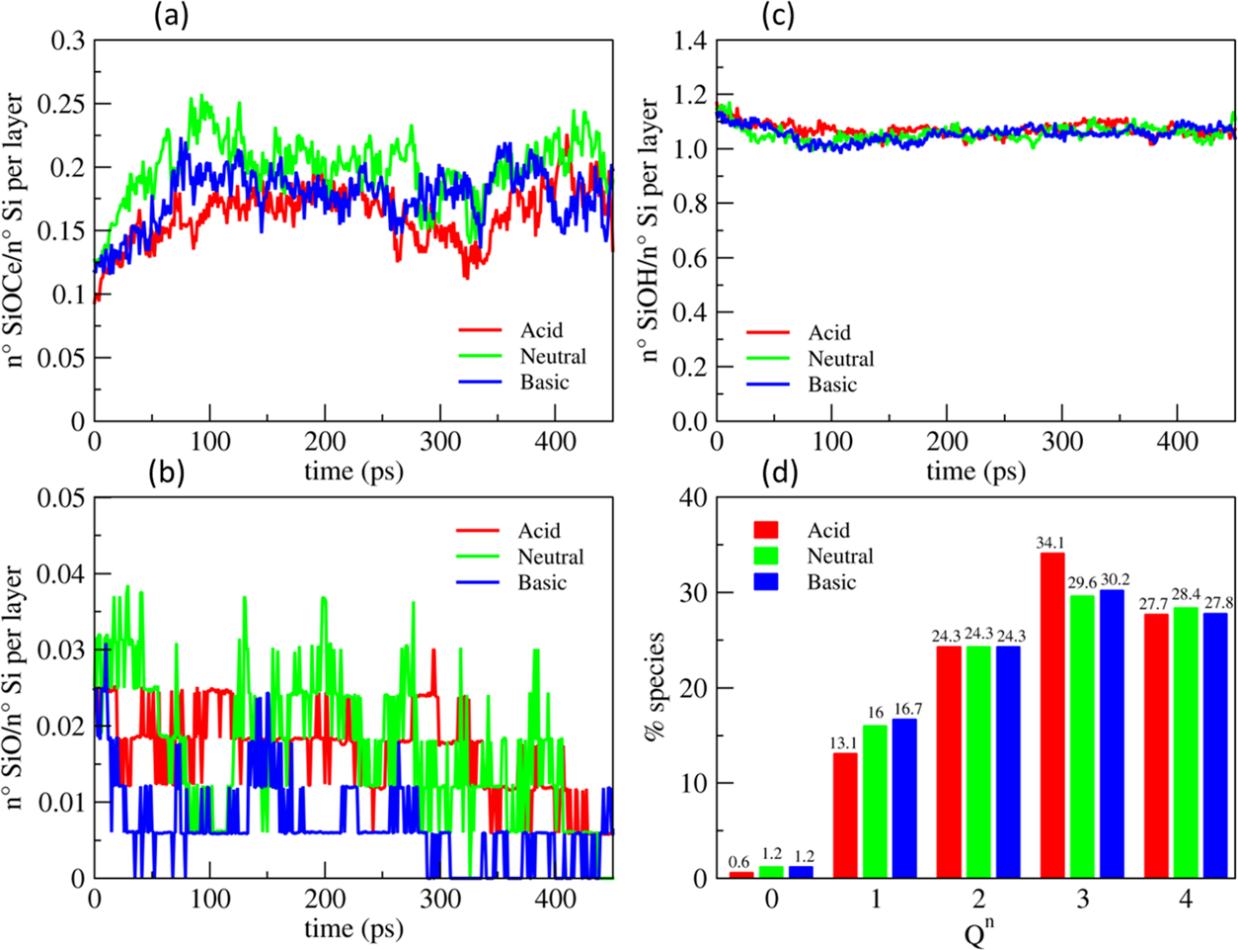
Evolution of the (a) Si–O–Ce bonds, (b) SiOH, and (c) NBOs at the interface between ceria (111) and the silica gel layer at 40% models (outermost 5 Å), both preconditioned in acidic, neutral, and basic solutions during the polishing simulation at 300 K and 5 GPa. (d) Comparison between the *Q^n^* distribution of silicon atoms in the outermost gel layers in the three models before and after polishing.

Figure 9a shows the time evolution of the amount of Si–O–Ce bonds per silicon atoms in the outermost layer during polishing as well as the *Q^n^* distributions of silicon atoms before and after polishing at different pH (acidic, neutral, and basic), maintaining *T* = 300 K and *P* = 5 GPa.

It is interesting to observe that the largest amount of Si–O–Ce bonds were formed at neutral pH, indicating the strongest interaction between the silica and ceria at this condition. This result agrees with the experimental evidence that more effective polishing can be achieved at this pH, which is due to the higher concentration of OH groups formed on the ceria (111) surface at neutral pH close to the IEP. The interaction should be weaker at basic pH since both the ceria and silica surfaces are negatively charged. Further, the interaction is even weaker at acidic pH because of the higher concentration of Ce–OH_2_^+^ species (molecularly bound water molecules) at the surface. In addition to the difference in the concentration of −OH groups, the Na^+^ and Cl^–^ ions located close to the interfaces might be another possible reason for reducing the number of Si–O–Ce bonds in the basic and acidic environments. The former, as observed above, is favorably absorbed on both the silica and ceria surfaces, which might hamper the direct interaction between the two surfaces to form the Si–O–Ce bridges.

The *Q^n^* distributions at the glass surface after polishing revealed that, in all cases, the degree of polymerization decreased with the detachment of two silicate units at neutral and basic pH and one unit at acidic pH. The degrees of silica network connectivity of the surfaces post-polishing were 2.75, 2.68, and 2.67, respectively. The surfaces polished at neutral and basic pH possessed a higher amount of *Q^n^* species with n ≤ 2. However, it must be highlighted that the *Q^n^* distributions do not significantly differ among the three cases for several reasons. First, the models studied are relatively small with respect to the real contact area between ceria and silica in experiments. Second, our sliding simulations were much shorter in timescale than the real process, which is often conducted for several minutes. During the experimental CMP process, the ceria nanoparticles frequently bump and slide on the silica substrate. Indeed, Cook^17^ estimated that one SiO_2_ molecule was removed for every twenty-four collisions between the ceria abrasive and the silica substrate. In this respect, it is not surprising that only 1 or 2 silicate units were removed during the MD simulations of only 450 ps.

##### 3.2.2.2 Effects of the Temperature

The temperature at the interface between a polisher and a substrate clearly affects the CMP process. The friction between the two surfaces may increase the local temperature up to the softening or melting points of the glass surface. An increase of temperature enhances the solubility of the silica to release the orthosilicic acid units and the reactions of the Si–O–Si bond dissociation and hydrolysis. Additionally, once the temperature increases close to the melting point of the glass; the surface may soften and lose the mechanical resistance against the polishing agent. Because the time frame of our simulations is too short to appropriately describe the heating of the system by the friction between the silica and ceria models, the temperature effects were evaluated by intentionally applying different temperatures, 300, 600, and 900 K. The simulations were performed using the Gel40% model in contact with the ceria (111) surface in neutral conditions and at 5 GPa.

**Figure 10:**
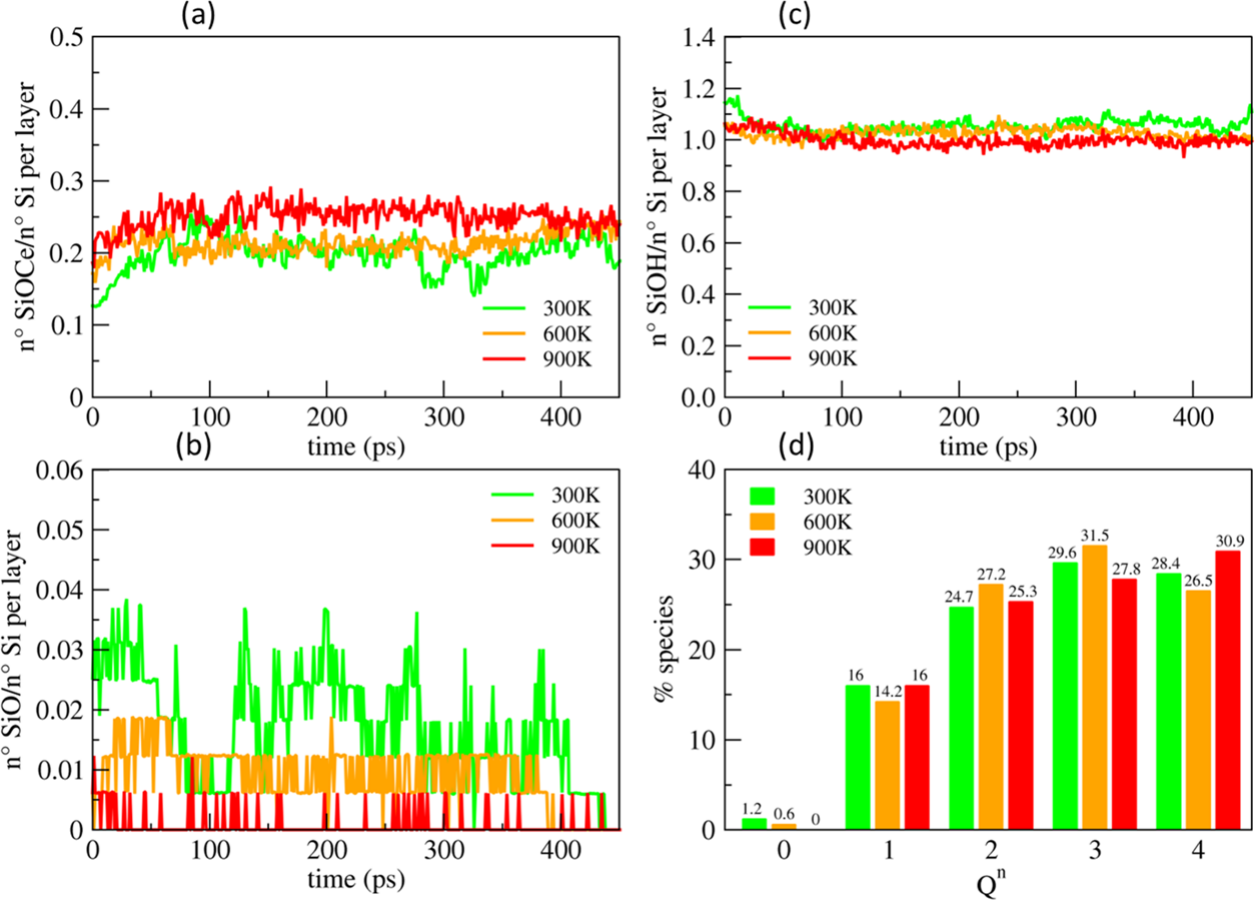
Evolution of the Si–O–Ce bonds (a), silanols (b), and NBOs (c) at the interface between ceria(111) and the silica gel layer at 40% models (outermost 5 Å), both preconditioned in the neutral solution during the polishing simulation at 5 GPa and temperatures of 300, 600, and 900 K. (d) Comparison between the *Q^n^* distribution of silicon atoms in the outermost gel layers in the three models before and after polishing.

Figure 10a shows that more Si–O–Ce bridges were formed with temperature increasing, resulting in a stronger interaction between the surfaces. At the highest temperature, 900 K, there were few NBOs on the silica surface since the silica surface oxygen was all involved in the Si–O–Ce bridges, as shown in Figure 10b. This is because the reactive Ce sites were increased by the hydrogen–hydroxyl recombination to release water from the ceria termination. Simultaneously, the higher temperature accelerated the dehydroxilation of the silica surface, which decreased the SiOH groups at the surface.

Note, however, that after the separation of the interfaces, the detachment of silica units was observed only at 300 K (2 units) and 600 K (1 unit), even though more Si–O–Ce bonds were formed at 900 K. Despite the increased reactivity between the surfaces, the high temperature seems to hinder the silica MRR. This might be ascribed to an increase of the competitive condensation reaction of the silanol pairs to form siloxane bridges. A simple index to recognize the condensation reaction is the number of *Q*^4^ species, and at 900 K, *Q*^4^ species increased from 28.4% (prepolishing reference, Figure 4d) to 30.9%, as shown in Figure 10d.

##### 3.2.2.3 Effect of the Pressure

**Figure 11:**
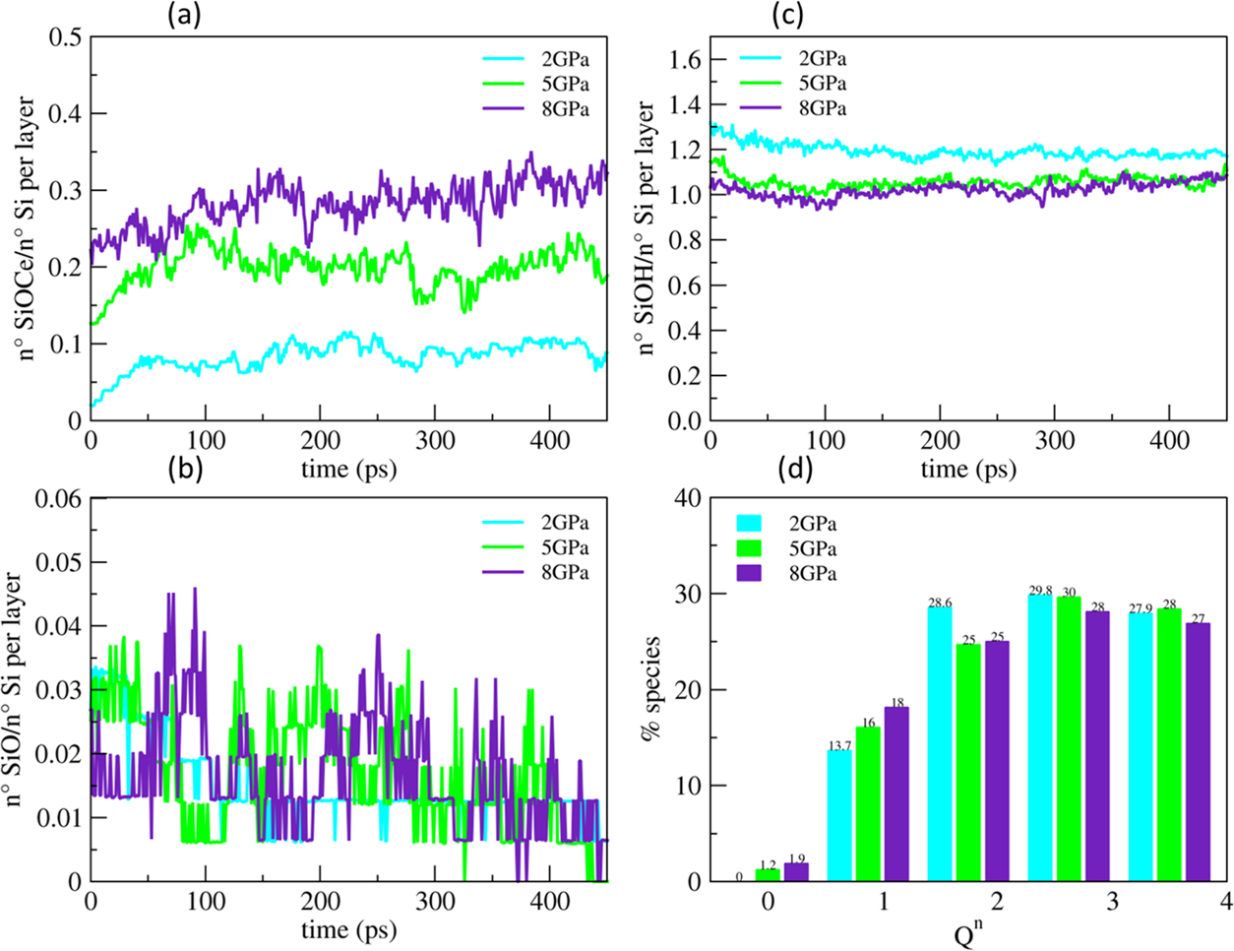
Evolution of the Si–O–Ce bonds (a), silanols (b), and NBOs (c) at the interface between ceria (111) and the silica gel layer at 40% models (outermost 5 Å), both preconditioned in a neutral solution during the polishing simulation at 300 K, under different applied pressure. (d) Comparison between the *Q^n^* distribution of silicon atoms in the outermost gel layers in the three models after polishing at different pressures.

To investigate the effect of the pressure on the CMP processes, the simulations were performed at 2, 5, and 8 GPa. As shown in Figure 11a, the higher pressure apparently increased Si–O–Ce bridges at the interface at the expense of the more silanols (Figure 11c). After the separation of the surfaces following the 450 ps of polishing simulations, the silicate units were detached at the pressures of 5 and 8 GPa. 2 units were detached at 5 GPa as *Q*_0_ species, while three units, a *Q*_0_ species and a dimer (Si_2_O_7_^6–^), were eliminated at 8 GPa. The connectivity of the silica network in the gel layer decreased more at the higher pressure after the polishing, as 2.72, 2.68, and 2.61 at 2, 5, and 8 GPa, respectively.

The higher compression clearly promoted stronger contact between the surfaces, which might improve the MRR of silica; however, there might be operative limits to the compression. For instance, at higher pressure, lumps of silica may be unexpectedly removed, and the surface becomes less smooth. Moreover, the high mechanical stress may induce undesired defects inside the glass substrate and produce cracks by breaking the siloxane networks.

#### 3.2.3 Effect of the Ceria Surface

**Figure 12:**
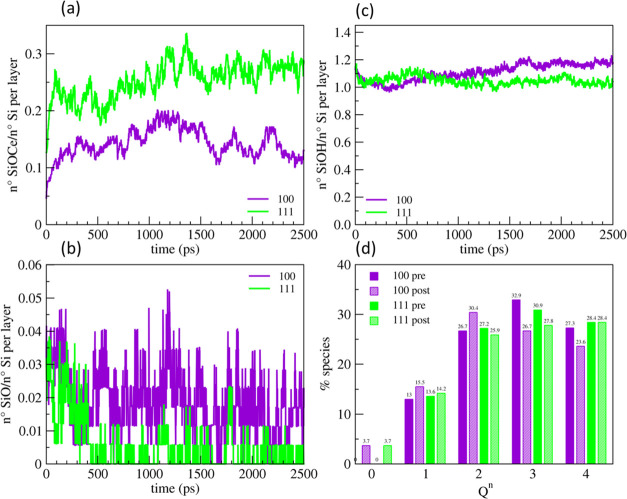
Evolution of the Si–O–Ce bonds (a), silanols (b), and NBOs (c) at the interface between silica gel 40% and the ceria surfaces (111) (in green) and (100) (in purple), preconditioned in the neutral solution during the polishing simulation at 300 K. (d) Comparison between the *Q^n^* distribution of silicon atoms in the outermost gel layers in the three models before and after polishing.

The ceria nanoparticles can be synthesized with different morphologies (nanorods, nanocubes, and nanospheres), in which not only the most stable (111) ceria surface but also the other low-index terminations can appear. For instance, polar (100) is known to be the dominant surface in the nanocubes.^9^ It is thus interesting to compare the mechanisms of silica extraction by the ceria surfaces (111) and (100). For a fair comparison, another silica gel 40% model whose surface area was fit to that of the ceria (111) model was built. It is worth noting that since the gel layers were generated by randomly removing a certain percentage of Si atoms from the 5 Å thick silica surface, there were slight differences in the *Q^n^* distribution and the silica network connectivity between the two Gel40% models, as shown in Figure 12d.

Figure 12 shows the time evolutions of the Si–O–Ce bonds (a), silanols (b), and NBOs (c) at the interfaces between the Gel40% models and the ceria surfaces (111) (in green) and (100) (in purple) during the polishing simulations at 300 K and 5 GPa. These MD simulations were conducted after preconditioning in the neutral solution. Figure 12d reports the *Q^n^* distributions of silicon atoms in the outermost gel layers of the two gel models after polishing. The polishing simulations were conducted longer, up to 2.5 ns, and the surface connectivity was analyzed after the separation of the interfaces.

The effects of the temperature, pressure, and pH on the effectiveness of the polishing with the ceria surface (100) were consistent with the case of the ceria surface (111); thus, the details are not reported. The time evolutions of the number of Si–O–Ce bonds, as shown in Figure 12a, demonstrated that more Si–O–Ce bonds (almost twice) per silicon atom were formed with the (111) surface, indicating that the (111) surface can more strongly interact with the silica gel than the (100) surface. This difference is explained by the different topologies in either the terminations or the surface density of Ce atoms between the two ceria surfaces. Indeed, the Ce atom density is 7.9 and 6.8 Ce per nm^2^ at the ceria (111) and (100) surfaces, respectively. Consequently, the (111) surface allows 3 Ce–O–Si bridges for a Si atom at maximum, while only a single Si–O–Ce bridge for a Si atom was observed on the ceria (100) surface.

**Figure 13:**
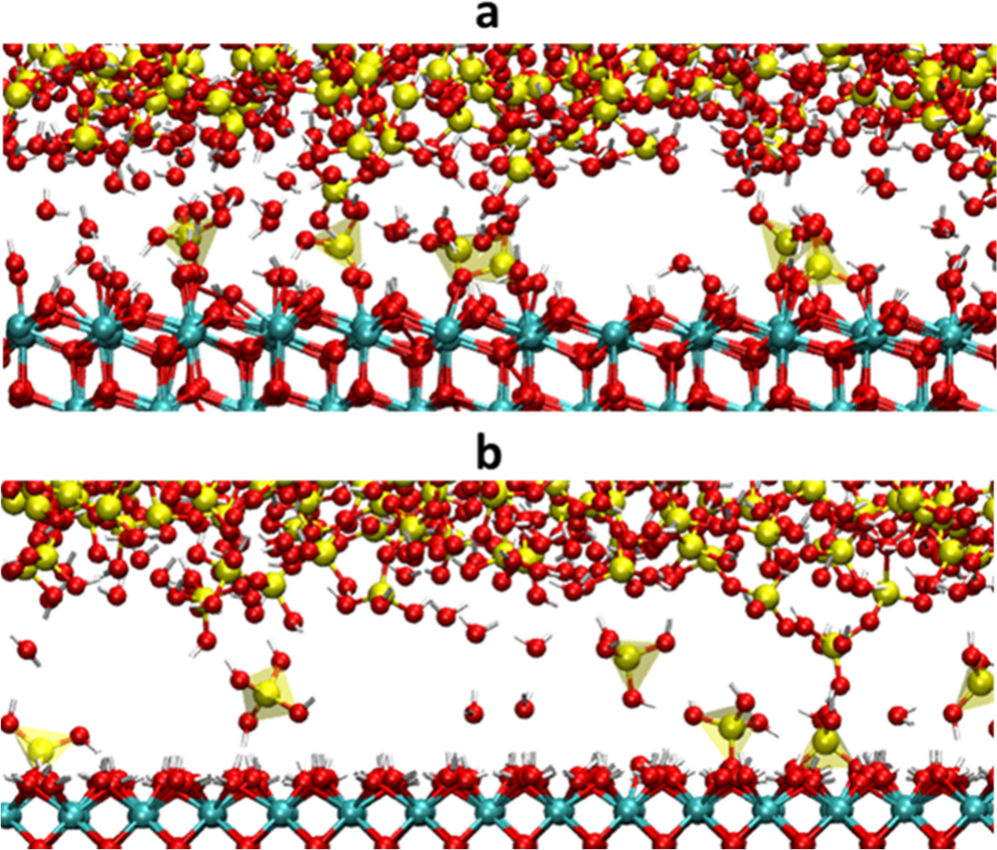
Separated interphases after 2.5 ns of polishing for the silica gel 40% with (a) ceria (111) and (b) ceria (100).

After 2.5 ns polishing, 6 silica units were detached from both models, as shown in Figure 13, indicating almost the same MRRs with the different ceria surfaces. This can be explained by the different reactivity of the two ceria surfaces.

Contrarily, the silica network connectivity of the gel layer decreased from 2.74 to 2.63 with ceria (111) and from 2.75 to 2.51 with ceria (100), implying that the ceria (100) surface is more effective in disrupting the silica network, even though the lower amount of Si–O–Ce bridges per surface units were formed. To seek the possible reason for the difference, the reaction energies to replace an adsorbed water molecule on the ceria surfaces by forming an orthosilicic acid molecule (H_4_SiO_4_) were calculated at the DFT/PBE0 level.
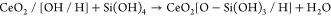
6

The reaction energies were almost the same (−0.65 and −0.47 eV on (111) and (100), respectively), while the binding energies of H_2_O and H_4_SiO_4_ molecules on the ceria surfaces were apparently different. The binding energies of a water molecule on the (111) and (100) surfaces were −0.50 and −2.20 eV, respectively. Those of a H_4_SiO_4_ molecule were −1.15 and −2.67 eV for the (111) and (100) surfaces, respectively. The higher binding energies on the (100) surface are ascribed to the lower coordination number of the Ce atoms, 6-coordinated, at the (100) surface in comparison with the sevenfold-coordinated Ce at the (111) surface. Note that fully coordinated Ce atoms in bulk CeO_2_ are eightfold-coordinated. Moreover, OH and O–Si(OH)_3_ formed the monodentate bonds with Ce atoms on the ceria (111) surface, while they formed the bidentate bonds with the Ce atoms on the ceria (100) surface. Therefore, although the ceria (100) abrasive forms fewer Si–O–Ce bridges with the silica substrate, the stronger bonding may facilitate the depolymerization of the silica network in comparison with the ceria (111) abrasive.

#### 3.2.4 Reaction Mechanisms of the Silica Removal

**Figure 14:**
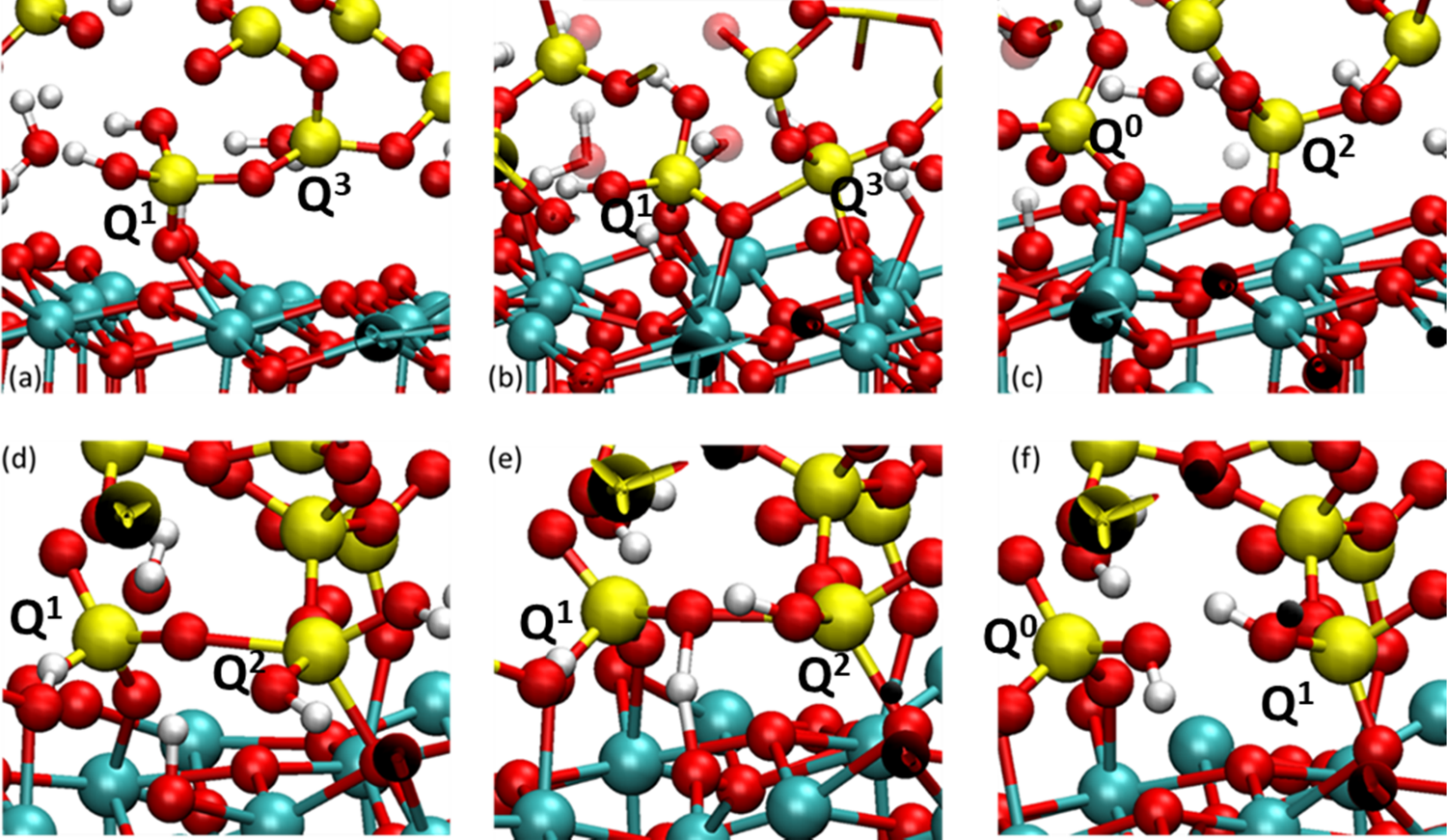
Possible reaction mechanisms of dissociation of siloxane bonds by the interaction of silica with CeO_2_ (111). (a) *Q*^1^ species bound to the ceria surface by one Si–O–Ce and to a *Q*^3^ species; (b) *Q*^1^ species is bonded to two Ce ions; the *Q*^3^ species expanded its coordination to five, thanks to the bond with surface oxygen of ceria and the bridging oxygen between the two silicate units bonds to a free Ce ion; (c) *Q*^0^ species is adsorbed on ceria; (d) *Q*^1^ (silicon on the left) and *Q*^2^ (right) species bonded to each other and on the ceria surface; (e) BO between the two *Q^n^* species is protonated by the Ce–OH group; (f) *Q*^0^ species formed thanks to the second mechanism is adsorbed on the ceria surface.

**Figure 15:**
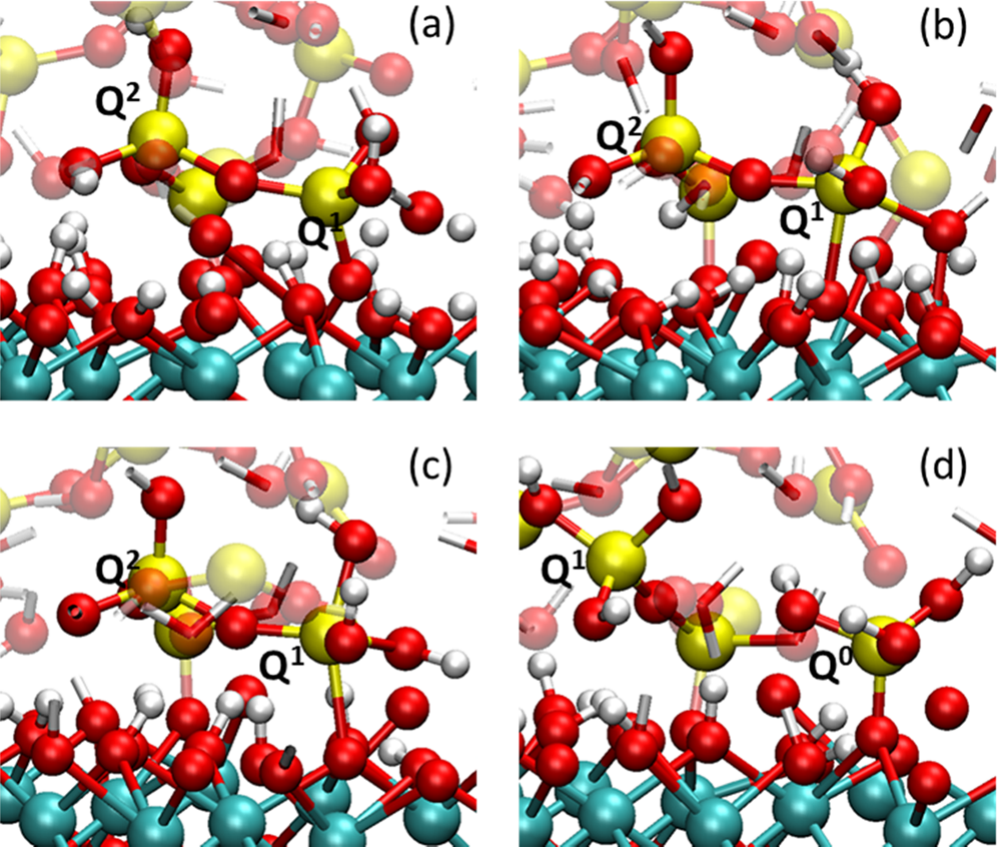
Possible reaction mechanism of dissociation of siloxane bonds by the interaction of silica with CeO_2_ (100). (a) On the right, a *Q*^1^ species bound to the ceria surface by one Si–O–Ce and to a *Q*^2^ species; (b) OH is transferred from the ceria surface to the *Q*^1^ species, which expands its coordination to five; (c) after about 20 ps from the formation of the 5-fold-coordinated silicon (on the right), it starts the elongation of the siloxane bond; (d) after the dissociation of the Si–O–Si bridge, *Q*^1^ (silicon on the left) and *Q*^0^ (right) species are formed, with *Q*^0^ attached to the ceria surface. The silica slab on which the silicate units are connected has been removed to simplify the figure.

According to the polishing simulations, three distinct mechanisms of the silica removals involving the dissociation of siloxane bridges were observed: two on ceria (111) and one on ceria (100). The reaction processes were visually reported in Figures 14 and 15, respectively.

##### 3.2.4.1 Ceria (111)

Figure 13 shows two types of reactions. In both mechanisms observed, the silica units with the different possible connectivities tightly interacted with the ceria surface by forming Si–O–Ce bridges between the Si–O groups and the Ce ions, and eventually, a bond was formed between Si and surface O on ceria. In the latter stage of this reaction, the Si atom extracted became fivefold-coordinated; thus, one of the siloxane bonds was stretched and, eventually, dissociated through mechanical wear by the sliding of the two surfaces. This mechanism is depicted in Figure 14a–c. Figure 14a shows that a *Q*^1^ species initially bonded to the ceria by forming a Si–O–Ce bond. This *Q*^1^ species was connected to a *Q*^3^ species, and it subsequently interacted with the ceria surface by forming a fivefold complex possessing an extra Si–O bond with a nearby O_S_ on the ceria surface (Figure 14b). Simultaneously, the BO connecting the *Q*^1^ and *Q*^3^ species bonded to the undercoordinated Ce site. The *Q*^1^ species was thus connected to two Ce atoms through two Si–O–Ce bonds. One of the BO–Si bonds of the *Q*^3^ species was considerably strained by the sliding process and dissociated after 4 ps. Therefore, the *Q*^1^ species turned into a *Q*^0^ species and remained on the ceria surface, whereas the *Q*^3^ species was transformed into the *Q*^2^ species connecting to the ceria surface through a Si–O–Ce bond.

The other mechanism of dissociation, shown in Figure 14d–f, involved the protonation of the BO between two silica units (*Q*^1^ species on the left and *Q*^2^ species on the right) by a proton donated by a Ce–OH group at the ceria surface. In this case, the siloxane bridge was immediately dissociated without the wear effect, contrary to the first mechanism.

##### 3.2.4.2 Ceria (100)

Because of the higher hydroxylation degree of the (100) termination (90%), most of the Si–O–Ce bridges were formed by replacing a hydroxyl on the ceria to bond with Si–O. The removal of the hydroxyl was promoted by its protonation from the Si–OH group and came closer to forming a water molecule. As for the first case on the (111) surface, the mechanism of the silica detachment involved the formation of a fivefold-coordinated silicon, but in this case, the extra coordination was formed with a hydroxyl donated from a vicinal Ce–OH site, as shown in Figure 15b, instead of an O_S_ atom. This intermediate lasted for approximately 20 ps, which was five times longer than the analogue on the ceria (111) surface, despite mechanical wear. Then, the extra siloxane bond dissociated following the elongation, which eventually provided *Q*^1^ species and *Q*^0^ species attached to the ceria surface, as depicted in Figure 15c,d.

## 4 Conclusions

Reactive molecular dynamics simulations were carried out using a new set of ReaxFF parameters, which was developed in this work for simulating the ceria/silica/water interactions to study the mechanisms of the interactions between ceria and the silica glass as well as the removal of small silicate units from silica glass by the frictions with ceria nanoparticles at the atomistic level.

The detailed reactive MD simulations on the CMP process revealed the following essential mechanisms. (1) The effect of the morphologies of the glass surfaces including the gel layers formed by hydroxylation of the surface was examined. Accordingly, the gelation of the silica surface was found to be crucial to form strong bonding between silica and ceria. (2) The reaction mechanisms of the silica extractions as an isolated SiO_4_ unit or dimers were studied by analyzing the time evolutions of the numbers of Si–O–Ce bonds and anchoring sites essential for the extraction. We visually showed several reaction paths to remove the silica units from the gel models. During the extraction, overcoordinated silicon appeared, and one of the Si–O–Si bonds was elongated and eventually disrupted to attach with Ce atoms. (3) The effects of pressure and temperature on the removal rate were examined. Basically, the higher temperature and pressure promoted the formation of Si–O–Ce bonds more, whereas the excessively high temperature proceeded the condensation reactions to reform the siloxane network in the gel layer, which lowered the polishing rate. This would be inconsistent with the experimental fact that the higher temperature basically accelerates polishing; however, our analysis indicated that the extremely high temperature due to overloading exhibits such an adverse effect. (4) The effect of the ceria surface morphology was studied by modeling (111) and (100) surfaces. Interestingly, the (111) surface can form more Si–O–Ce bonds, while the Ce atoms at the (100) surface can make stronger bidentate bonds with silicon because of the lower coordination. The stronger binding at the (100) surface compensated for the fewer Si–O–Ce bonds compared with the (111) surface, which eventually resulted in almost the same removal rates. This may be one of the possible reasons for the more efficient polishing with the (100) surface in the real CMP process.

The other open questions remaining are related to the effect of the defects, which are oxygen vacancies and Ce^3+^ ions at the ceria surface and the subsurface. Their effects must be investigated at the quantum mechanical level of calculations. Finally, the CMP process should be modeled with realistic models of ceria nanoparticles with different morphology (nanorods, nanospheres, and nanocubes) instead of using flat surfaces.

Despite this, the work sheds light on the atomic chemical and mechanical details of the CMP process for the silica substrates using the ceria nanoparticles and provides a hint for efficient polishing by designing the slurry compositions and chemistry as well as by optimizing the morphology of the ceria nanoparticles.

## Supporting Information Available

Detailed description of the quantum mechanical calculations performed to generate the dataset on which the reactive force-fields parameters have been parametrized as well as the strategy to optimize the parameters (PDF)
Library file for the elements Si/O/Ce/H/Na/Cl in the format suitable for the LAMMPS code (TXT)
Coordinates in the cif format of the silica surface models used in the simulations (ZIP)
