# Supplementary material for: New Atomistic Insights on the Chemical Mechanical Polishing of Silica Glass with Ceria Nanoparticles
Source: Langmuir. 2023 Apr 8;39(15):5527–41. doi: 10.1021/acs.langmuir.3c00304 (PMC10116594; doi:10.1021/acs.langmuir.3c00304)
Supplement: Supplementary file 1 — la3c00304_si_001.pdf [file la3c00304_si_001.pdf]

Supporting Information for

New Atomistic Insights on the Chemical Mechanical Polishing of Silica Glass with Ceria Nanoparticles

Luca Brugnoli,[a] Katsuaki Miyatani,[b] Masatoshi Akaji,[c] Shingo Urata,[b] and Alfonso Pedone[a*]

[a)]Department of Chemical and Geological Sciences, University of Modena and Reggio Emilia, via G. Campi 103, 41125, Modena, Italia

[b)]Innovative Technology Laboratories, AGC Inc., Yokohama, Kanagawa 230-0045, Japan

c) Electronics Company, AGC Inc., Yokohama, Kanagawa 230-0045, Japan

*corresponding author email: alfonso.pedone@unimore.it

Extension of the Ce/O/H ReaxFF library to Silicon: the Ce-Si and Ce-O-Si interactions.

The training set adopted to fit the parameters for the Ce-Si and Ce-O-Si interactions was formed by the DFT optimized crystalline structures as well as the bulk modulus and elastic constants of the cerium silicate $CeSiO_4$[1,2]; the reaction energy of condensation of a $Si(OH)_4$ unit on the CeO_2 (111) and CeO_2 (100) hydroxylated surfaces; the potential energy surface for the detachment of a $Si(OH)_4$ unit on the surface (100) and the potential energy surface for the bending of the Ce-O-Si angle for the orthosilicic acid adsorbed on CeO_2(100). The structures included in the training are shown in **figure S1**.

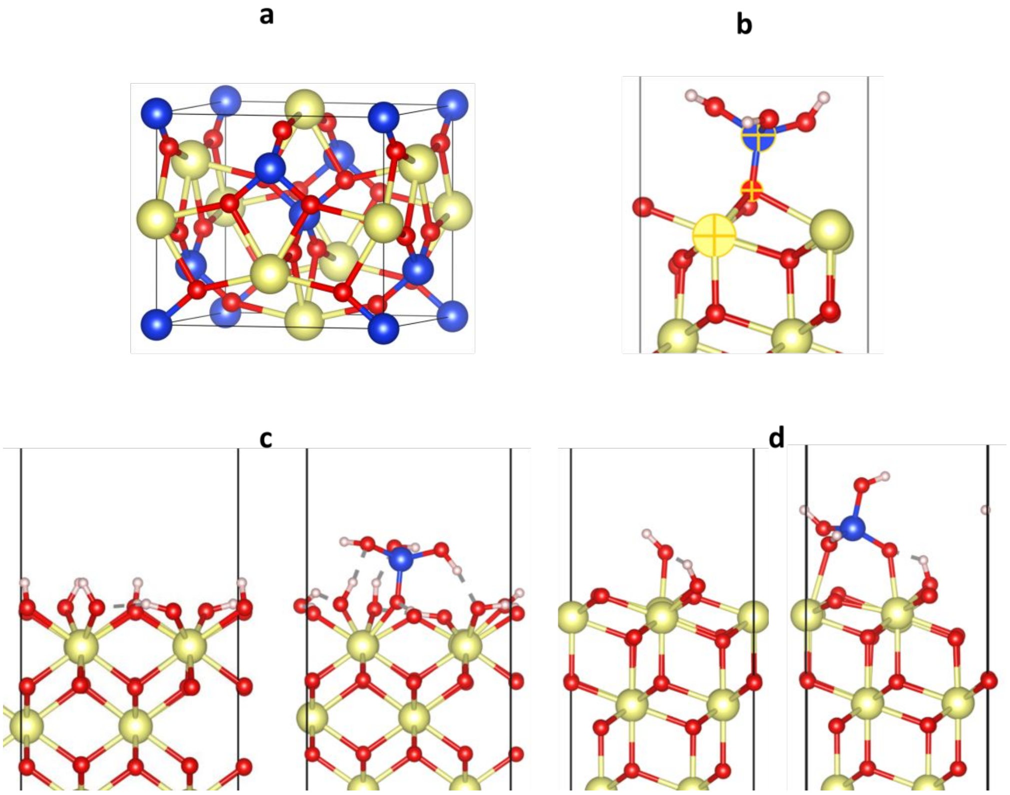

Figure S1: Structural models included in the fitting: (a) Crystalline structure of $CeSiO_4$; (b) (111) CeO_2 surface with a orthosilicic unit used to fit the PES of the Si-O-Ce angle; (c) fully hydroxylated CeO_2 (100) surface with an $Si(OH)_4$ unit replacing a water molecule used to fit the relative reaction energy; (d) CeO_2 (111) partially hydroxylated with a $Si(OH)_4$ replacing a water molecule. Yellow, blue, red and white spheres are respectively Ce, Si, O and H atoms.

The GULP code[3] has been used to optimize the parameters through the minimization of the cost function (F) reported in **eq 1** :

$$F = \sum_i^n \sigma_i (X_{i,QM} - X_{i,ReaxFF})^2, \qquad i = 1,2,3 \ldots n \qquad (1)$$

Where the X_i is the value of the *i*-th observable computed at the QM or ReaxFF level, σ_i is the weight value assigned to each data point. As a rule of thumb, the weights assigned to the variables are inversely proportional to the error typically associated with the associated observable, thus fractional coordinates and lattice parameters, typically more accurate, have higher weights, while relative energies and elastic properties have lower weights. In the present works, the weighs assigned to fractional coordinates and lattice parameters are respectively 10^4 and 10^3; for relative energies as reaction energies and the Si-O-Ce potential energy surface the weights are 10^2, while for the bulk modulus and the elastic constants were used values of 10^0 and 10^{-1},

respectively. The parameters of the two and three body interactions Ce-Ce and Ce-O-Ce developed in our previous work[4] where used as starting values in the fitting of the Ce-Si and Ce-O-Si parameters.

All DFT calculations were performed with the code CRYSTAL17, which employs atom-centered Gaussian functions as basis sets. The Global hybrid PBE0 functional[5] was adopted in virtues of its rather good reproduction of the main properties of interest of both cerium oxides CeO_2 and Ce_2O_3, of the reduced phases CeO_{2-x}, as well as amorphous silica, α-quartz and silicates.[6–10]

As in previous works, two distinct basis sets BS1 and BS2 were adopted depending on the task. In both basis sets, Ce inner core was described by a semi-relativistic effective core potential (ECP),[11] while the valence electrons $4s^2 4p^6 5s^2 4d^{10} 5p^6 6s^2 5d^1 4f^1$ were described by the contraction scheme (10sp8d8f)/[4sp3d3f] in BS1, and by the contraction scheme (12s12p9d8f)/[8s7p4d4f] in BS2. As for O, two distinct basis were used in BS1 for the cerium oxide and for the molecular species as H_2O and $Si(OH)_4$: an all electron 8-411(d) basis set with the outer exponents optimized for cerium oxides[7] were used for the O in ceria surfaces, while the larger basis described by the contraction scheme (13s7p2d4f)/[7s4p7p2df] was adopted in the other cases. The same larger O contraction was used for all the O species in BS2. In both BS1 and BS2, H and Si were described by a POB-TZVP all-electron basis set.[12]

The smaller basis set BS1 was used in the optimization of the ceria surfaces with dissociated water molecules and $Si(OH)_4$, while the larger and more accurate BS2 was used to computed energies from single points calculations on the structures optimized with BS1. Instead, all the calculations on cerium silicates were carried out adopting the BS2 basis set.

The experimental crystalline structure of $CeSiO_4$[1,2] (space group I41/amd) reported in Figure 1 was taken as starting point for the DFT optimization. The reaction of water replacement by $Si(OH)_4$ shown in **eq 2**:

$$CeO_2\,/[OH/H] + Si(OH)_4 \rightarrow CeO_2\,[O\text{-}Si(OH)_3/H] + H_2O \qquad (2)$$

was computed on the ceria surfaces 111 and 100.

For CeO_2 (111) a supercell p (2 x 2), formed by 9 atomic layers ($Ce_{12}O_{24}$), was cleaved from the optimized bulk (a = 5.401 Å), while for CeO_2 (100) it was adopted a supercell c (2 x 2), formed by 13 atomic layers ($Ce_{24}O_{48}$), reconstructed by shifting half of the raw O in terminal layer to the opposite Ce terminated layer.

The reciprocal space of $CeSiO_4$ was sampled adopting a Monkhorst-Pack grid[13] 4 × 4 × 4. For the 2D surface models of CeO_2 (111) and (100), instead, it was used a k-point mesh of 4 x 4 x 1. The Gauss-Legendre radial quadrature and Lebedev two-dimensional angular point distribution schemes were adopted to generate the integration grid.[14] The default grid (XLGRID) was employed for all calculations.

In CRYSTAL, the truncation criteria of the Coulomb and exchange infinite lattice series are controlled by five thresholds (T1, T2, T3, T4, and T5): these have been set to 10^{-8} (T1−T4) and 10^{-16} (T5) for all the calculations (see the referenced manual[15] of the software for further details). The convergence of the self-consistent field was considered achieved when difference in energy between consecutive steps was below 10^{-7} Hartree, in geometry optimization and single point calculations, and 10^{-8} Hartree in the elastic properties calculations. The optimization of the structure was considered achieved when both the maximum atomic gradient and maximum atomic displacement were simultaneously below 0.00045 Hartree/Bohr and 0.0012 Bohr, respectively.

The fitted parameters (the complete Ce/O/H/Si/Na/Cl library is reported at the end of the ESI) reproduced reasonably well the crystalline structure and the elastic properties of the cerium silicates as shown in **Table S1** considering that the only parameters fitted involved the Ce-Si pairs and the Ce-O-Si tree-body terms, without modifying the original parameters for the other Ce/O/H and Si/O/H interactions in order to maintain consistency.

Another set of observables fitted, the potential energy surface for the angle θ of Si-O-Ce of a O-$Si(OH)_3$ group bound on the surface CeO_2 (111), is rather well reproduced by the ReaxFF as shown in **Figure S2**. The PES computed at the ReaxFF shows two minima at θ = 100 and 140°, due to hydrogen bond formation between the hydroxyls and the surface O of the oxide, while the QM PES shows a single minima for θ = 120 °, without H bonds.

Finally, the reaction of replacement of a water molecule by an orthosilicic unit (eq. 2) on the surfaces (111) and (100) computed at the ReaxFF level are -0.54 and -0.46 eV, respectively in good agreement with the DFT values of -0.65 and -0.47 eV.

Table S1: Comparison between the theoretical observables of the structural and elastic properties of $CeSiO_4$ and the ones resulting from the fit. The available experimental data are reported.

		**ReaxFF**	**DFT**	**Exp.**
$CeSiO_4$	a (Å)	7.119	6.980	6.9746[1]
	c (Å)	6.496	6.226	6.2055[1]
	B_0 (GPa)	171.2	180.0	-
	C_{11} (GPa)	260.8	301.2	-
	C_{12} (GPa)	79.4	57.2	-
	C_{13} (GPa)	153.6	136.4	-
	C_{33} (GPa)	386.6	402.7	-
	C_{44} (GPa)	41.8	71.0	-

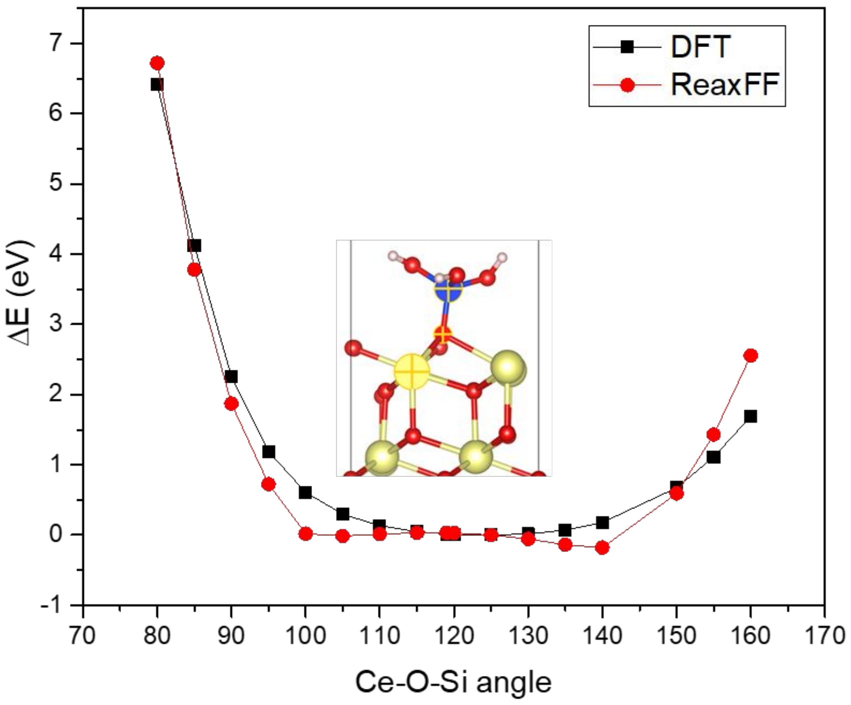

Figure S2: Comparison of the PES of the angle Ce-O-Si on the (111) surface computed at the DFT and ReaxFF level.

References

(1) Schlüter, J.; Malcherek, T.; Husdal, T.; Andersen. The New Mineral Stetindite, CeSiO4, a Cerium End-Member of the Zircon Group. *Neues Jahrb. Für Mineral. - Abh.* **2009**, *186* (2), 195–200. https://doi.org/10.1127/0077-7757/2009/0146.

(2) Estevenon, P.; Kaczmarek, T.; Vadot, F.; Dumas, T.; Solari, P. L.; Welcomme, E.; Szenknect, S.; Mesbah, A.; Moisy, P.; Poinssot, C.; Dacheux, N. Formation of $CeSiO_4$ from Cerium(III) Silicate Precursors. *Dalton Trans.* **2019**, *48* (28), 10455–10463. https://doi.org/10.1039/C9DT01990A.

(3) Gale, J. D. GULP: A Computer Program for the Symmetry-Adapted Simulation of Solids. *J. Chem. Soc. Faraday Trans.* **1997**, *93* (4), 629–637. https://doi.org/10.1039/A606455H.

(4) Brugnoli, L.; Menziani, M. C.; Urata, S.; Pedone, A. Development and Application of a ReaxFF Reactive Force Field for Cerium Oxide/Water Interfaces. *J. Phys. Chem. A* **2021**. https://doi.org/10.1021/acs.jpca.1c04078.

(5) Adamo, C.; Barone, V. Toward Reliable Density Functional Methods without Adjustable Parameters: The PBE0 Model. *J. Chem. Phys.* **1999**, *110* (13), 6158–6170. https://doi.org/10.1063/1.478522.

(6) Da Silva, J. L. F.; Ganduglia-Pirovano, M. V.; Sauer, J.; Bayer, V.; Kresse, G. Hybrid Functionals Applied to Rare-Earth Oxides: The Example of Ceria. *Phys. Rev. B* **2007**, *75* (4). https://doi.org/10.1103/PhysRevB.75.045121.

(7) Graciani, J.; Márquez, A. M.; Plata, J. J.; Ortega, Y.; Hernández, N. C.; Meyer, A.; Zicovich-Wilson, C. M.; Sanz, J. Fdez. Comparative Study on the Performance of Hybrid DFT Functionals in Highly Correlated Oxides: The Case of CeO_2 and Ce_2O_3. *J. Chem. Theory Comput.* **2011**, *7* (1), 56–65. https://doi.org/10.1021/ct100430q.

(8) Brugnoli, L.; Ferrari, A. M.; Civalleri, B.; Pedone, A.; Menziani, M. C. Assessment of Density Functional Approximations for Highly Correlated Oxides: The Case of CeO_2 and Ce_2O_3. *J. Chem. Theory Comput.* **2018**, *14* (9), 4914–4927. https://doi.org/10.1021/acs.jctc.8b00600.

(9) Demichelis, R.; Civalleri, B.; Ferrabone, M.; Dovesi, R. On the Performance of Eleven DFT Functionals in the Description of the Vibrational Properties of Aluminosilicates: Performance of Eleven DFT Functionals. *Int. J. Quantum Chem.* **2010**, *110* (2), 406–415. https://doi.org/10.1002/qua.22301.

(10) Plascencia, C.; Curtiss, L. A.; Liu, C. Hydrogen Activation by Silica-Supported Metal Ion Catalysts: Catalytic Properties of Metals and Performance of DFT Functionals. *J. Phys. Chem. A* **2019**, *123* (1), 171–186. https://doi.org/10.1021/acs.jpca.8b08340.

(11) Dolg, M.; Stoll, H.; Savin, A.; Preuss, H. Energy-Adjusted Pseudopotentials for the Rare Earth Elements. *Theor. Chem. Acc. Theory Comput. Model. Theor. Chim. Acta* **1989**, *75* (3), 173–194.

(12) Oliveira, D. V.; Laun, J.; Peintinger, M. F.; Bredow, T. BSSE-Correction Scheme for Consistent Gaussian Basis Sets of Double- and Triple-Zeta Valence with Polarization Quality for Solid-State Calculations. *J. Comput. Chem.* **2019**, *40* (27), 2364–2376. https://doi.org/10.1002/jcc.26013.

(13) Monkhorst, H. J.; Pack, J. D. Special Points for Brillouin-Zone Integrations. *Phys Rev B* **1976**, *13*, 5188–5192.

(14) Towler, M. D.; Zupan, A.; Causà, M. Density Functional Theory in Periodic Systems Using Local Gaussian Basis Sets. *Comput. Phys. Commun.* **1996**, *98* (1), 181–205. https://doi.org/10.1016/0010-4655(96)00078-1.

(15) R. Dovesi, V. R. Saunders, C. Roetti, R. Orlando, C. M. Zicovich-Wilson, F. Pascale, B. Civalleri, K. Doll, N. M. Harrison, I. J. Bush, P. D'Arco, M. Llunell, M. Causà, Y. Noël, L. Maschio, A. Erba, M. Rerat and S. Casassa. CRYSTAL17 User's Manual (University of Torino, Torino, 2017), 2017.
